# Spectroscopic Studies on Photoinduced Reactions of the Anticancer Prodrug, *trans,trans,trans*‐[Pt(N_3_)_2_(OH)_2_(py)_2_]

**DOI:** 10.1002/chem.201705349

**Published:** 2018-02-05

**Authors:** Robbin R. Vernooij, Tanmaya Joshi, Michael D. Horbury, Bim Graham, Ekaterina I. Izgorodina, Vasilios G. Stavros, Peter J. Sadler, Leone Spiccia, Bayden R. Wood

**Affiliations:** ^1^ School of Chemistry and Centre for Biospectroscopy Monash University Clayton 3800 VIC Australia; ^2^ Department of Chemistry University of Warwick Gibbet Hill Road Coventry CV4 7AL UK; ^3^ Institute of Radiopharmaceutical Cancer Research Helmholtz-Zentrum Dresden-Rossendorf 01328 Dresden Germany; ^4^ Monash Institute of Pharmaceutical Sciences Monash University Parkville VIC 3052 Australia

**Keywords:** anticancer agents, attenuated total reflection, mechanism of action, Pt^IV^ prodrugs, vibrational spectroscopy

## Abstract

The photodecomposition mechanism of *trans,trans,trans*‐[Pt(N_3_)_2_(OH)_2_(py)_2_] (**1**, py=pyridine), an anticancer prodrug candidate, was probed using complementary Attenuated Total Reflection Fourier Transform Infrared (ATR‐FTIR), transient electronic absorption, and UV/Vis spectroscopy. Data fitting using Principal Component Analysis (PCA) and Multi‐Curve Resolution Alternating Least Squares, suggests the formation of a *trans*‐[Pt(N_3_)(py)_2_(OH/H_2_O)] intermediate and *trans*‐[Pt(py)_2_(OH/H_2_O)_2_] as the final product upon 420 nm irradiation of **1** in water. Rapid disappearance of the hydroxido ligand stretching vibration upon irradiation is correlated with a −10 cm^−1^ shift to the antisymmetric azido vibration, suggesting a possible second intermediate. Experimental proof of subsequent dissociation of azido ligands from platinum is presented, in which at least one hydroxyl radical is formed in the reduction of Pt^IV^ to Pt^II^. Additionally, the photoinduced reaction of **1** with the nucleotide 5′‐guanosine monophosphate (5′‐GMP) was comprehensively studied, and the identity of key photoproducts was assigned with the help of ATR‐FTIR spectroscopy, mass spectrometry, and density functional theory calculations. The identification of marker bands for some of these photoproducts (e.g., *trans*‐[Pt(N_3_)(py)_2_(5′‐GMP)] and *trans*‐[Pt(py)_2_(5′‐GMP)_2_]) will aid elucidation of the chemical and biological mechanism of anticancer action of **1**. In general, these studies demonstrate the potential of vibrational spectroscopic techniques as promising tools for studying such metal complexes.

## Introduction

Platinum therapy is standard practice for approximately 50 % of patients undergoing anticancer treatment, but suffers from major drawbacks.^1^ These include acquired and inherent resistance as well as off‐target effects leading to short‐ and long‐term strain on the patient.^2^ Similar shortcomings for other chemotherapeutic agents in clinical use have stimulated the search for new anticancer drugs exhibiting novel mechanism(s) of action.

Photoactivatable metal‐based prodrugs can release their reactive metal‐based species locally upon activation by light, also referred to as photoactivated chemotherapy (PACT). It has been shown in the past decade that such metal‐based PACT candidates can provide localised toxicity through their metal core and/or their released ligands.^3^

**Figure 1:**
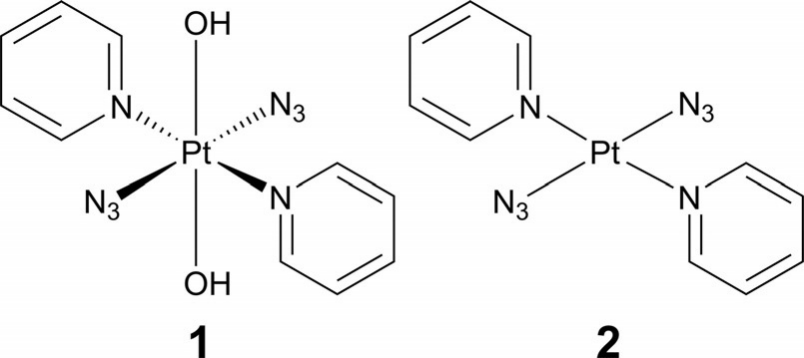
Molecular structures of *trans*,*trans*,*trans*‐[Pt(N_3_)_2_(OH)_2_(py)_2_] (**1**) and its synthetic precursor *trans*‐[Pt(N_3_)_2_(py)_2_] (**2**).

*Trans,trans,trans*‐[Pt(N_3_)_2_(OH)_2_(py)_2_] (**1**, py=pyridine, Figure 1) is one such potent PACT prodrug candidate capable of providing reactive Pt(IV/II)‐, azido‐ and hydroxyl‐ (radical) based species.^4^ Complex **1** can be specifically activated locally in cancer cells by UVA, blue or green light, affording a multitargeted biological activity, whilst being inert and non‐toxic in the dark.^4^, ^5^ Recently, we reported a comprehensive vibrational spectroscopic study of **1**, identifying its highly defined mid‐ and far‐IR vibrational fingerprints.^6^ In order to provide new insight into our understanding of the mechanism of action of **1**, we now study the photodecomposition of **1** using Attenuated Total Reflection Fourier Transform Infrared (ATR‐FTIR) spectroscopy.^6^, ^7^ Additionally, the photoinduced reaction of **1** with guanosine 5′‐monophosphate (5′‐GMP) was investigated. Transient Electronic Absorption Spectroscopy (TEAS) has been employed to investigate the ultrafast photodynamics of **1**, with UV/Vis spectroscopy and mass spectrometry being used to study the photoinduced reactions and for identification of the potential photoproducts.^4^, ^8^

Data analysis has been performed using Principal Component Analysis (PCA) and Multi‐Curve Resolution Alternating Least Squares (MCR‐ALS), which allow for correlation of data from the various techniques. Moreover, interpretation of the captured spectral changes has been supported with Density Functional Theory (DFT) calculations, aiding the assignment of the observed dynamics.

## Results

### Photodecomposition of 1 by ATR‐FTIR

**Figure 2:**
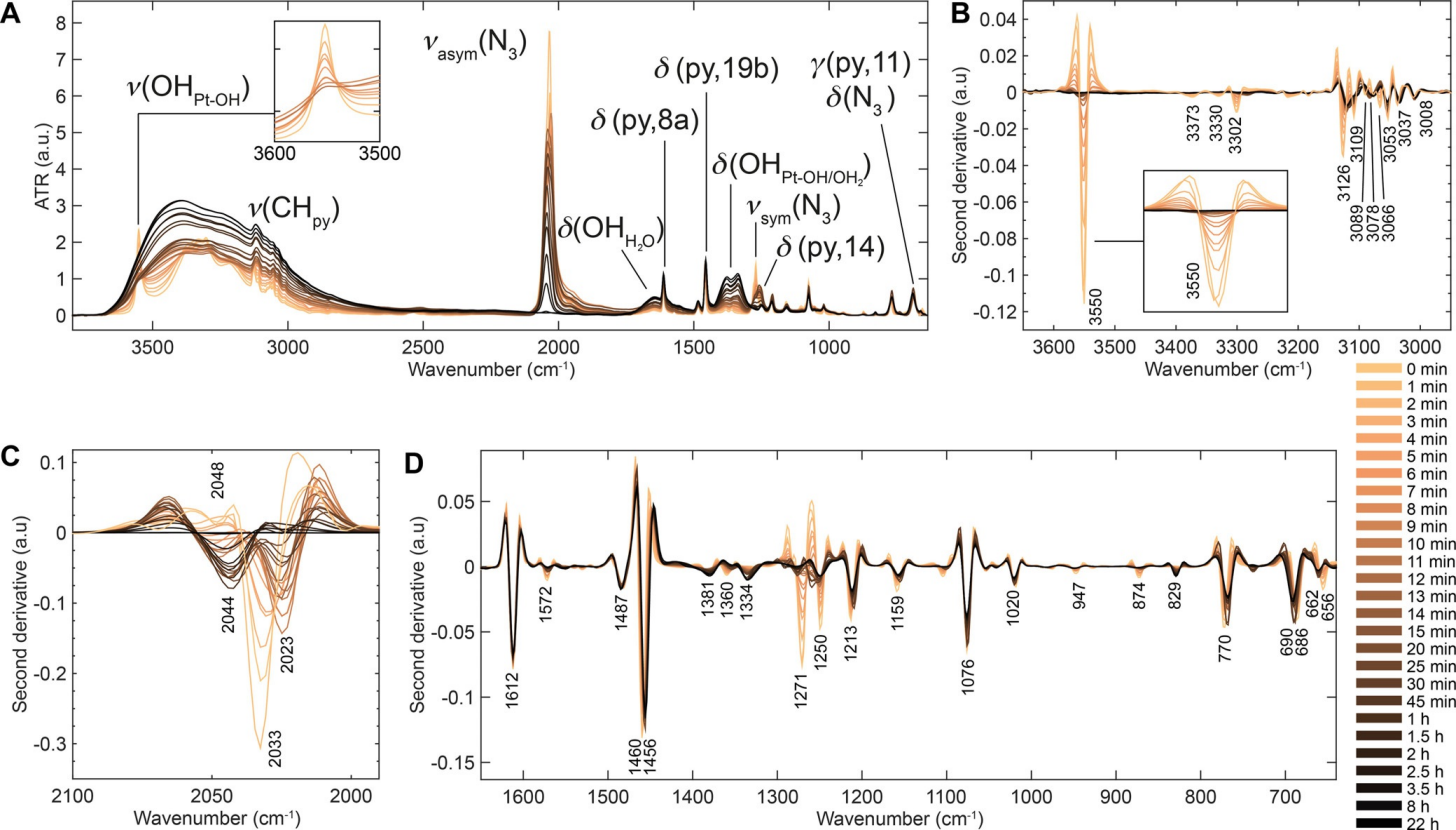
ATR‐FTIR spectra of the photodecomposition of **1** (2 mm in water) under 420 nm irradiation. A) Full spectral range, 3700–640 cm^−1^. B) Second derivative spectra, 3650–2950 cm^−1^, highlighting rapid decrease in *ν*(OH_Pt‐OH_) at 3350 cm^−1^ in the first few minutes. C) Second derivative spectra, 2100–1980 cm^−1^, highlighting the decrease and shifts in *ν*_sym_(N_3_) at 2048/2033 cm^−1^ to 2023 cm^−1^ and 2044 cm^−1^ sequentially prior to complete disappearance. D) Second derivative spectra, 1650–640 cm^−1^, highlighting decrease in *ν*_sym_(N_3_) at 1271 cm^−1^ and increase in *δ*(OH_Pt‐OH_) at 1381 cm^−1^ and 1334 cm^−1^. Notations used: *ν*=stretch, *δ*=in‐plane angle bending, *γ*=out‐of‐plane angle bending, sym=symmetric and asym=antisymmetric. The labelling of pyridine modes is given in Wilson's notations, as described previously.^9^

Studies of the photodecomposition of **1** under 420 nm irradiation, monitored by ATR‐FTIR, were carried out as described in the Experimental section. The evolution of the ATR‐FTIR spectra of the full spectral range between 3700–640 cm^−1^ is shown in Figure 2 A, which includes selected characteristic peak assignments for **1** reported previously.^6^ The detection of changes in Figure 2 A is aided by the corresponding second derivative spectra divided into three spectral windows, 3650–2950 cm^−1^, 2100–1980 cm^−1^, and 1650–640 cm^−1^ (Figure 2 B–D).

The first spectral window between 3650 and 2950 cm^−1^, Figure 2 B, shows a single sharp hydroxido stretching vibration, *ν*(OH_Pt‐OH_), at 3550 cm^−1^, three azido overtones (3373 cm^−1^, 3330 cm^−1^, and 3302 cm^−1^), pyridine CH stretching vibrations *ν*(CH_py_) between 3126 cm^−1^ and 3008 cm^−1^, and a *ν*(OH) band centred at approximately 3300 cm^−1^, which is very broad due to hydrogen bonding. Upon 420 nm irradiation, a rapid decrease in *ν*(OH_Pt‐OH_) is observed in the first 10 min (Figure 2 B) in which complete disappearance is reached after 20 min (Figure S2 in the Supporting Information, intensity vs. time plot), whilst the broad *ν*(OH) feature increases from the start to the end (Figure 2 A). Concurrently, the *ν*(CH_py_) vibrations broaden and shift slightly (<5 cm^−1^) over time. Furthermore, slow removal of the azido overtones is observed.

The second spectral window between 2100 cm^−1^ and 1980 cm^−1^ covers the antisymmetric azido stretching vibration, *ν*_asym_(N_3_). Prior to irradiation, *ν*_asym_(N_3_) consists of one main vibration at 2033 cm^−1^ and a shoulder at 2048 cm^−1^ as indicated in the second derivative spectra in Figure 2 C. During the first 5 min of irradiation, a decrease in intensity of both of these bands is observed and additionally the main vibration at 2033 cm^−1^ shifts to 2023 cm^−1^. Continued irradiation shifts the main vibration to 2044 cm^−1^ prior to complete removal (Figure 2 C).

The third spectral window between 1700 cm^−1^ and 640 cm^−1^ contains several pyridine ring deformations and CH stretching vibrations (1612, 1572, 1487, 1460, 1381, 1360, 1250, 1213, 1159, 1076, 1020, 847, 874, 770, 686, and 665 cm^−1^) as assigned previously, such as *δ*(py,8a) at 1612 cm^−1^ (Figure 2 A,D).^6^ Furthermore, it contains the symmetric azido stretching vibrations, *ν*_sym_(N_3_), at 1271 cm^−1^ and its shoulder at 1278 cm^−1^, which overlap with the pyridine bending mode, *δ*(py,14), at 1250 cm^−1^. Additionally, the azido bending mode, *δ*(N_3_), at 686 cm^−1^ is observed which overlaps with the out‐of‐plane bending vibration of pyridine (*γ*(py,11), Figure 2 A, D).

As shown in Figure 2 A and D, there is little effect on the pyridine vibrations throughout the irradiation process. Minor shifts do occur as shown by the gradual shift of the pyridine bending mode, *δ*(py,19b), from 1460 cm^−1^ to 1456 cm^−1^ (Figure 2 D). The removal of the azido vibrations can be observed by the changes to *ν*_sym_(N_3_) and *δ*(N_3_), but is less dramatic compared to the antisymmetric stretching vibration. Both these vibrations overlap with pyridine modes, *δ*(py,14) and *γ*(py,11), respectively, which remain present at 1250 cm^−1^ and 690 cm^−1^ after completion of the photodecomposition as shown in Figure 2 D.

Interestingly, a broad band gradually appears upon irradiation underneath the two previously assigned weak pyridine bending modes at 1381 cm^−1^ and 1360 cm^−1^ (*δ*(py, 3), in‐ and out‐of‐phase) (Figure 2 A,D), with the peak at 1360 cm^−1^ shifting to 1334 cm^−1^. This broad band can be reasonably assigned to the OH bending mode of bound water to platinum (*δ*(OH${}_{\mathrm{Pt}\text{-}\mathrm{OH}/\mathrm{OH}{}_{2}}$ )) and is considered in more detail in the Discussion section, below. At the same rate, a broad water bending mode, *δ*(OH_H2O_), appears at ≈1650 cm^−1^ (Figure 2 A and Figure S3 for spectrum of water vs. photodecomposition of **1**).

The presence of water in the resulting product matrix is clearly indicated by the growth of the broad *ν*(OH) and *δ*(OH) bands of water centred at ≈3300 cm^−1^ and ≈1650 cm^−1^ (Figure 2 A). Furthermore, a new OH bending mode is observed underlying the pyridine bending modes at 1381 cm^−1^ and 1334 cm^−1^ (Figure 2 A, D).

Part of the experimental procedure involves the evaporation of solvent by a N_2_ flow directed onto the ATR crystal prior to measurement by ATR‐FTIR (see Experimental Section). When comparing spectra taken with the N_2_ flow left on during measurement, the intensity and band shapes of the *ν*(OH) and *δ*(OH) bands are reduced (Figure S4). The new band that appears underlying the pyridine bending modes at 1381 cm^−1^ and 1334 cm^−1^ is also influenced by the nitrogen flow, however, its intensity is increased, whereas all other bands remain unchanged. Additionally, when 1 scan (≈1 s) ATR‐FTIR measurements are recorded continuously, whilst turning the N_2_ flow ON and OFF with two second intervals, the spectra interconvert without intermediates visible (Figure S4).

### ATR‐FTIR: PCA

Principal component analysis (PCA) was carried out in order to capture the major trends in the data and correlate the observed spectral changes. PCA is an unsupervised analytical data method to assess large data sets and is widely used in a variety of sciences and industries.^10^ In PCA, a data set is reduced in dimensionality by finding directions through the data set where the variation is greatest. The first principal component (PC) accounts for the majority of variation in the sample spectra and each successive orthogonal PC accounts for a decreasing proportion of the variance. Each spectrum can ultimately be represented as a point in multivariate space plotted on the PCs. In the case of the ATR‐FTIR data set, each PC represents a new axis that the projected objects (scores) can be plotted against each other in order to find patterns or clusters, that is, how samples are correlated or anti‐correlated. The PC loadings are the cosine angles that the scores make with the centroid and can be analysed to determine what variables (wavenumber values) are responsible for the clustering.

**Figure 3:**
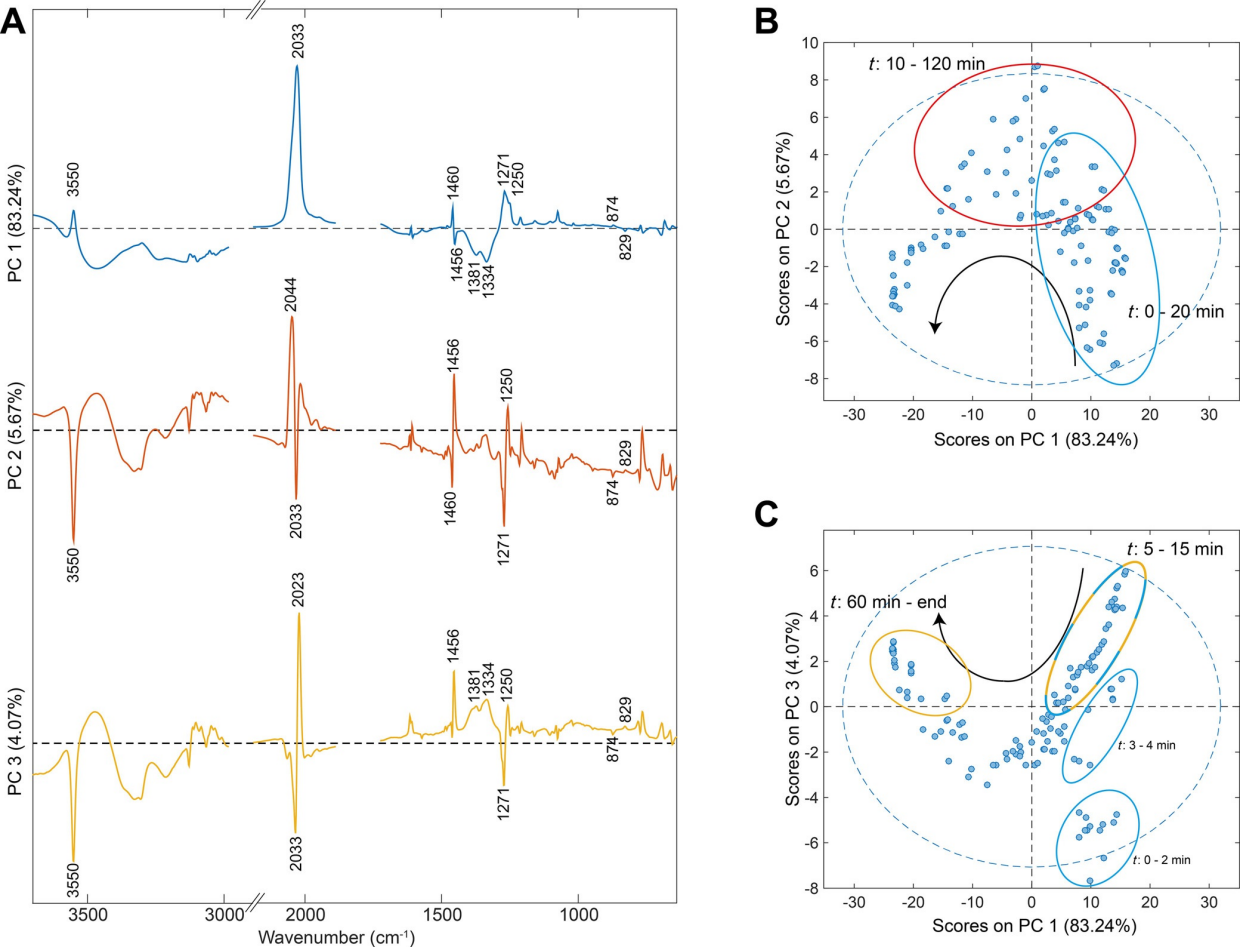
PCA scores and loading plots for the photodecomposition of **1** under 420 nm irradiation by ATR‐FTIR. A) Loading plots of PC1 (blue), PC2 (red) and PC3 (yellow). Black dashed line=0, spectral regions were cut for the sake of clarity after PCA analysis. B) PC1 vs. PC2. C) PC1 vs. PC3. Black arrows indicate time trajectory in score plots, ellipse encircle specified time points and are coloured according to the positive PC score, blue dashed line=95 % confidence limit.

The full spectral region (3700–650 cm^−1^) was used to carry out the PCA analysis on the photodecomposition of **1** under 420 nm irradiation by ATR‐FTIR and three PCs were selected, capturing 93 % of the variance. The resulting PCA scores and loading plots are shown in Figure 3; individual loading plots and PC vs. time plots are in Figure S5 in the Supporting Information. In this case, positive scores are associated with positive loadings and vice versa. The PCA loading vibrations and irradiation times with positive PC scores are tabulated in Table 1, and are described below.

**Table 1:** Selected PCA loading vibrations and irradiation times with positive PC scores.

PC	*ν*(OH_Pt‐OH_)^[a]^	*ν*_asym_(N_3_)^[a]^	*ν*_sym_(N_3_)^[a]^	*δ*(py,19b)^[a]^	*δ*(OH${}_{\mathrm{Pt}\text{-}\mathrm{OH}/\mathrm{OH}{}_{2}}$ )^[a]^	*γ*(py, 10a)^[a]^	*t* [min]
1	3550 +	2033 +	1271 +	1640 +	1381 −	874 +	0–4, 15–20 PC1
				1456 −	1334 −	829 −	5–15 PC1+3
							
2	3550 −	2044 +	1271 −	1640 −		874 −	10–120 PC2
		2033 −		1456 +		829 +	
							
3	3550 −	2033 −	1271 −	1640 −	1381 +	874 −	5–15 PC1+3
		2023 +		1456 +	1334 +	829 +	60–end PC3

[a] Unit=cm^−1^, + or − indicate positive or negative PCA loading.

Both score plots PC1 vs. PC2 and PC1 vs. PC3, follow a trajectory over time as indicated by the black arrow in Figure 3 B and C. Positive scores for PC1 are observed mostly from 0 to 20 min (Figure 3 B,C), with highest scores in the first 4 min. After 5 min of irradiation, a combination of positive scores is obtained for PC1 and PC3, reducing towards 15 min (Figure 3 C, blue and yellow ellipse). It continues to transition into PC2, which remains dominant until 120 min of irradiation (Figure 3 B). Subsequently, the scores transition back into PC3, which continues to become more prominent until the end of irradiation (Figure 3 C). Due to the positive scores on both PC1 and PC3 from 5 to 15 min (Figure 3 C, blue/yellow circle), it can be deduced confidently, with the related loadings, that this is due to an initial shift in the *ν*_asym_(N_3_) of −10 cm^−1^ (2033 to 2023 cm^−1^) combined with the reduction of *ν*(OH_Pt‐OH_) at 3550 cm^−1^ (Figure 3 A, blue and yellow). This feature is strongly present after 5 min and reduces towards 15 min. Further transition of the *ν*_asym_(N_3_) is then captured in PC2, in which the main peak is located at 2044 cm^−1^ (Figure 3 A,B). Thus, a second shift of *ν*_asym_(N_3_) is captured by the PCA of +21 cm^−1^ after removal of *ν*(OH_Pt‐OH_) (2023 to 2044 cm^−1^). Positive scores on PC2 remain present until 120 min of irradiation, hereafter PC3 becomes dominant until the completion of the photodecomposition of **1** (Figure 3 C). This captures the growth of the new broad *δ*(OH${}_{\mathrm{Pt}\text{-}\mathrm{OH}/\mathrm{OH}{}_{2}}$ ) band (1381 and 1334 cm^−1^) from 60 min onwards. The PCA analysis further captures more gradual changes to the spectra, such as the shift of δ(py,19b) from 1460 cm^−1^ to 1456 cm^−1^, as well as the −45 cm^−1^ shift of the γ(py, 10a) vibration from 874 to 829 cm^−1^.

### Photodecomposition of 1 by HPLC

Analytical HPLC (254/214 nm) was carried out in order to identify the amounts of species formed throughout the photodecomposition of **1**. HPLC traces were recorded for samples taken for ATR‐FTIR at selected time points, and are shown in Figure S6. Starting from complex **1** at time zero, two new products appear after 5 min with different retention times (P1 and P2) prior to the appearance of another absorption P3, which remains as the final product. Products P1 and P2 evolve at the same ratio based on the integrated HPLC peak areas (ratio P2**/**P1=0.43±0.09, between 1–60 min), which indicate that one of these species could be the result of solvent exchange (Figure S7).

### Photodecomposition of 1 by UV/Vis

The evolution of photoproducts formed during the photodecomposition of **1** was further investigated by UV/Vis spectroscopy. The UV/Vis spectrum of **1** (50 μm, water) in the dark prior to irradiation was in line with a previous report (Figure S8).^4^ It contains two characteristic absorption bands at 294 nm and 260 nm, assigned as LMCT (N_3_, OH→Pt; LMCT=ligand‐to‐metal charge‐transfer) and mixed ^1^LMCT/^1^IL (OH→Pt,N_3_; IL=interligand) transitions.^4^ The decrease in intensity of the absorption band at 294 nm upon irradiation was monitored to determine the pseudo quantum yields.^4^ Here, the trends and species formed upon irradiation have been investigated, which has not been carried out previously. Figure S8 shows the photodecomposition of **1** (50 μm) under 420 nm irradiation by UV/Vis, where the absorption band at 294 nm disappears whilst the band at 260 nm increases and shifts to 256 nm in the first 10 min before decreasing with no further changes observed after 150 min.

The photodecomposition of **1** and its synthesised precursor, *trans*‐[Pt(N_3_)_2_(py)_2_] (**2**, Figure 1), under 310 nm irradiation in acetonitrile were additionally examined by UV/Vis. The same evolution for **1** was observed regardless of the solvent or excitation wavelength (Figure S13). The UV/Vis spectrum of **2** (50 μm, acetonitrile) in the dark prior to irradiation contains two characteristic absorption bands at 330 nm and 263 nm, with another weak band at 403 nm observable at higher concentrations (Figure S12). Upon 310 nm irradiation, both the 330 nm and 263 nm absorption bands decrease over time, including a blue shift of 11 nm in the band at 263 nm to 252 nm at completion (Figure S14). Overlaying the photodecomposition time points 10 and 4 min of **1** and **2** in acetonitrile reveal a high degree of similarity (Figure S15).

### ATR‐FTIR and UV/Vis: multi‐curve fitting

Multi‐Curve Resolution Alternating Least Squares (MCR‐ALS) was carried out to analyse the photodecomposition of **1** and **2**, as described in the Experimental Section. The UV/Vis data were fitted using a two‐step kinetic model, capturing the observed changes in the spectra (Figure S8, S13, and S14 in the Supporting Information). The resulting fitted spectra and concentration profiles, including rate constants are shown in Figure S16. Fitted spectra of the photoproducts are labelled P1+P2 and P3 for **1** based on the analytical HPLC traces and P1 and P2 for **2**. In all three instances, the first trace is alike to the corresponding dark spectra of **1** in water as well as acetonitrile, and **2** in acetonitrile (Figure S16 A,C,E, respectively). The first intermediate product of **1** in water under 420 nm irradiation has two absorption bands at 315 nm and 256 nm (P1+P2), with the final product (P3) containing a single absorption band at 256 nm. The photodecompositions of **1** and **2** in acetonitrile under 310 nm irradiation both show identical spectra for their first intermediate species (**1**: P1+P2 and **2**: P1) with absorption bands at 297 nm and 254 nm. Their corresponding final products (**1**: P3 and **2**: P2) contain a single absorption band at 253 nm and 252 nm, respectively.

Using the same kinetic model, the ATR‐FTIR spectra between 2100–640 cm^−1^ of the photodecomposition of **1** in water under 420 nm irradiation were fitted by MCR‐ALS and photoproduct assignments can be proposed based on the deconstructed ATR‐FTIR spectra (Figure S17 in the Supporting Information). The deconstructed spectrum of the photodecomposition of **1** at 0 min is in line with the dark spectrum (Figure 2 A) with the main *ν*_asym_(N_3_) vibration at 2031 cm^−1^. The P1+P2 species has a single *ν*_asym_(N_3_) vibration centred a 2042 cm^−1^, the *δ*(OH${}_{\mathrm{Pt}\text{-}\mathrm{OH}/\mathrm{OH}{}_{2}}$ ) bands at 1379 cm^−1^ and 1334 cm^−1^ and all corresponding *trans* pyridine modes as outlined in Figure 2 A.

### TEAS: photodecomposition of 1

TEAS studies of **1** in acetonitrile using 310 nm UVA excitation pulses were carried out to gain insight into the ultrafast photodynamics occurring prior to the formation of the observed species by steady state ATR‐FTIR, HPLC, and UV/Vis. Attempts using blue 420 nm excitation in both water and acetonitrile resulted in no observable transient species.

**Figure 4:**
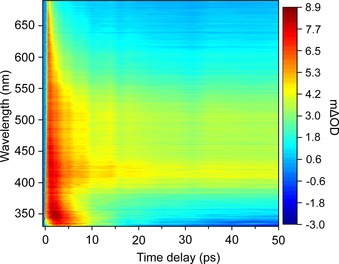
TAS of **1** in acetonitrile (2 mm) upon 310 nm excitation, displayed as a false colour map, indicating the change in optical density (mΔOD).

The transient absorption spectra (TAS) are presented as a false colour‐map, shown in Figure 4. After initial excitation, the TAS are dominated by a broad excited state absorption (ESA) feature spanning 350–690 nm. As the pump–probe time delay (Δ*t*) increases (≈1 ps), the ESA begins to undergo two spectral changes, consisting of a decay in the ESA at wavelengths longer than 550 nm and a growth in absorption at wavelengths shorter than 350 nm. This evolution leads to a broad sloped ESA spanning the entire probe window. As Δ*t* further increases, three (positive) features begin to emerge, around 345 nm, 420 nm and 500 nm. At Δ*t* >5 ps the peak at 345 nm begins to decay away to reveal a negative spectral feature by ≈20 ps, leaving the ESA peaks at 420 nm and 500 nm, which extends to the maximum available Δ*t* (2 ns). The resulting negative feature is assigned to a ground state bleach (GSB) as it overlaps with the ground state absorption of **1**.

**Figure 5:**
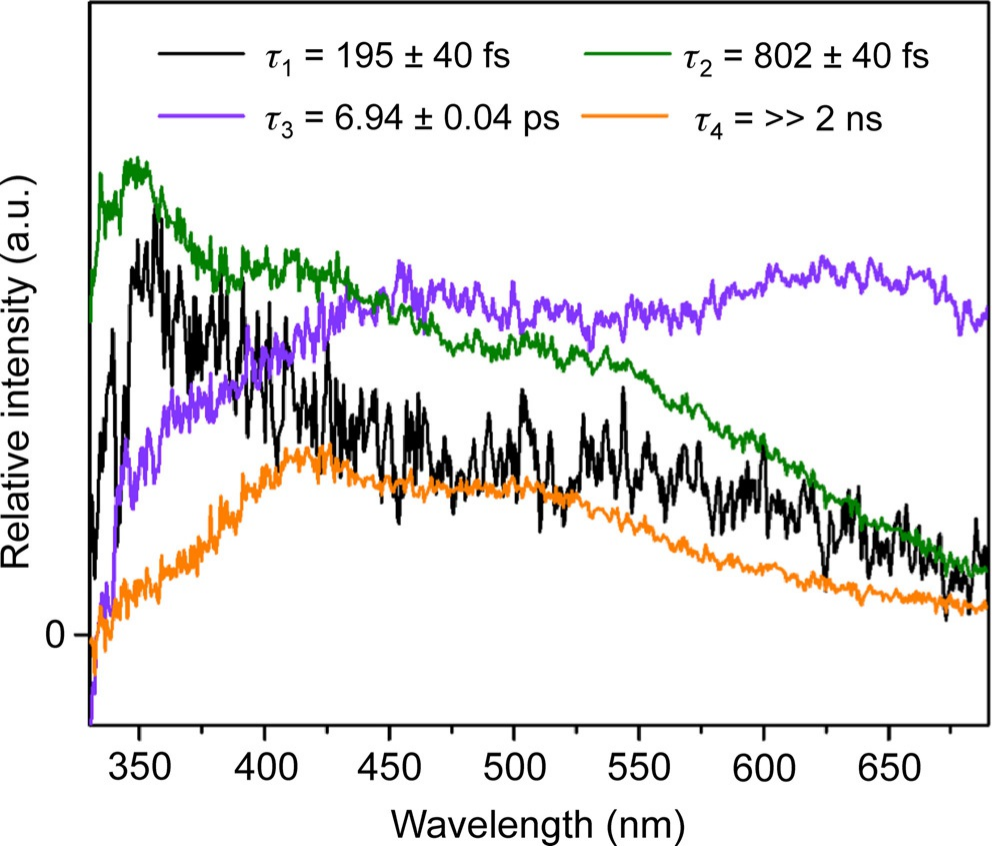
EADS from the sequential global fitting analysis of the TAS data for **1** in acetonitrile (2 mm) upon 310 nm excitation, with their associated time‐constants.

To extract the dynamical information in the TAS, a sequential global fitting analysis was carried out using the Glotaran software package.^11^ To fully model the TAS, four time‐constants (*τ_n_*=*k_n_* ^−1^) were used and the fit was convoluted with the instrument response function (≈80 fs). The resulting evolution‐associated difference spectra (EADS) and corresponding time‐constants are shown in Figure 5. We will discuss the significance of these EADS in more detail later (see Discussion). We note that the value given for *τ*_4_ was significantly greater than the maximum available experimental Δ*t*, thus quoted as ≫2 ns. The TAS residual plot, which reports on the goodness of fit, can be found in the Supporting Information (Figure S18).

### ATR‐FTIR: photoinduced reaction of 1 with 5′‐GMP

The photoinduced reaction of **1** with 5′‐GMP monitored by ATR‐FTIR under 420 nm irradiation was carried out as described in the Experimental Section. Results are divided into the ATR‐FTIR spectra of compounds in the dark and the photoinduced reaction spectra between 3800–1950 cm^−1^, 1800–1200 cm^−1^ and 1190–640 cm^−1^.

#### 1 and 5′‐GMP prior to irradiation

The dark ATR‐FTIR spectra of 5′‐GMP and **1**+5′‐GMP were investigated prior to irradiation. The experimental bands for 5′‐GMP and the DFT‐calculated infrared frequencies of 5′‐GMP match the experimental data (Table S1, Figure S1 in the Supporting Information). These assignments are in line with previous infrared and Raman reports of 5′‐GMP, guanosine, and guanine.^12^ Several characteristic vibrations of both **1** and 5′‐GMP independently are retained upon mixing **1** with 5′‐GMP (2 mol equiv) as shown in Figure S1. In the 3800–2500 cm^−1^ region, the *ν*(OH_Pt‐OH_) of **1** (3550 cm^−1^) is split up into four peaks at 3591 cm^−1^, 3568 cm^−1^, 3550 cm^−1^ and 3512 cm^−1^ with the broad featured *ν*(OH) of 5′‐GMP at ≈3300 cm^−1^ underlying those vibrations. Furthermore, the *ν*_asym_(NH_2_) and *ν*_sym_(NH_2_) at 3321 cm^−1^ and 3113 cm^−1^, respectively, of 5′‐GMP can be observed (Figure S1). A minor shift is observed for the main *ν*_asym_(N_3_) vibration of **1** from 2033 cm^−1^ to 2031 cm^−1^. The 1800–645 cm^−1^ region mainly contains 5′‐GMP vibrations, yet, several vibrations of **1** are still present. These include, *δ*(py,8a) at 1612 cm^−1^ (combined with 1610 cm^−1^ of 5′‐GMP), *δ*(py,19b) at 1460 cm^−1^, *ν*_sym_(N_3_) at 1271 cm^−1^, *δ*(py,9a) at 1076 cm^−1^ (combined with 1078 cm^−1^ of 5′‐GMP), *γ*(py,4) at 770 cm^−1^ (combined with 777 cm^−1^ of 5′‐GMP) and *γ*(py,11)+*δ*(N_3_) at 690 cm^−1^ (Figure S1). A range of combination bands from 5′‐GMP are observed between 1800–1485 cm^−1^, which are primarily purine‐ring‐related CO, CC, and CN stretching vibrations plus NH/NH_2_ bending modes, and are considered in more detail during the irradiation experiments below. Further 5′‐GMP vibrations in the 1800–645 cm^−1^ region consist of the purine breathing modes at 1377 cm^−1^, 1360 cm^−1^, and 1178 cm^−1^. The 5′‐GMP phosphate vibrations are located at 1076 cm^−1^ (*ν*_asym_(PO_3_^2−^, broad), 976 cm^−1^ (*ν*_sym_(PO_3_^2−^) and 802/777 cm^−1^ (*ν*(PO)).

#### Photoinduced reaction of 1 with 5′‐GMP (3800–1950 cm^−1^)

**Figure 6:**
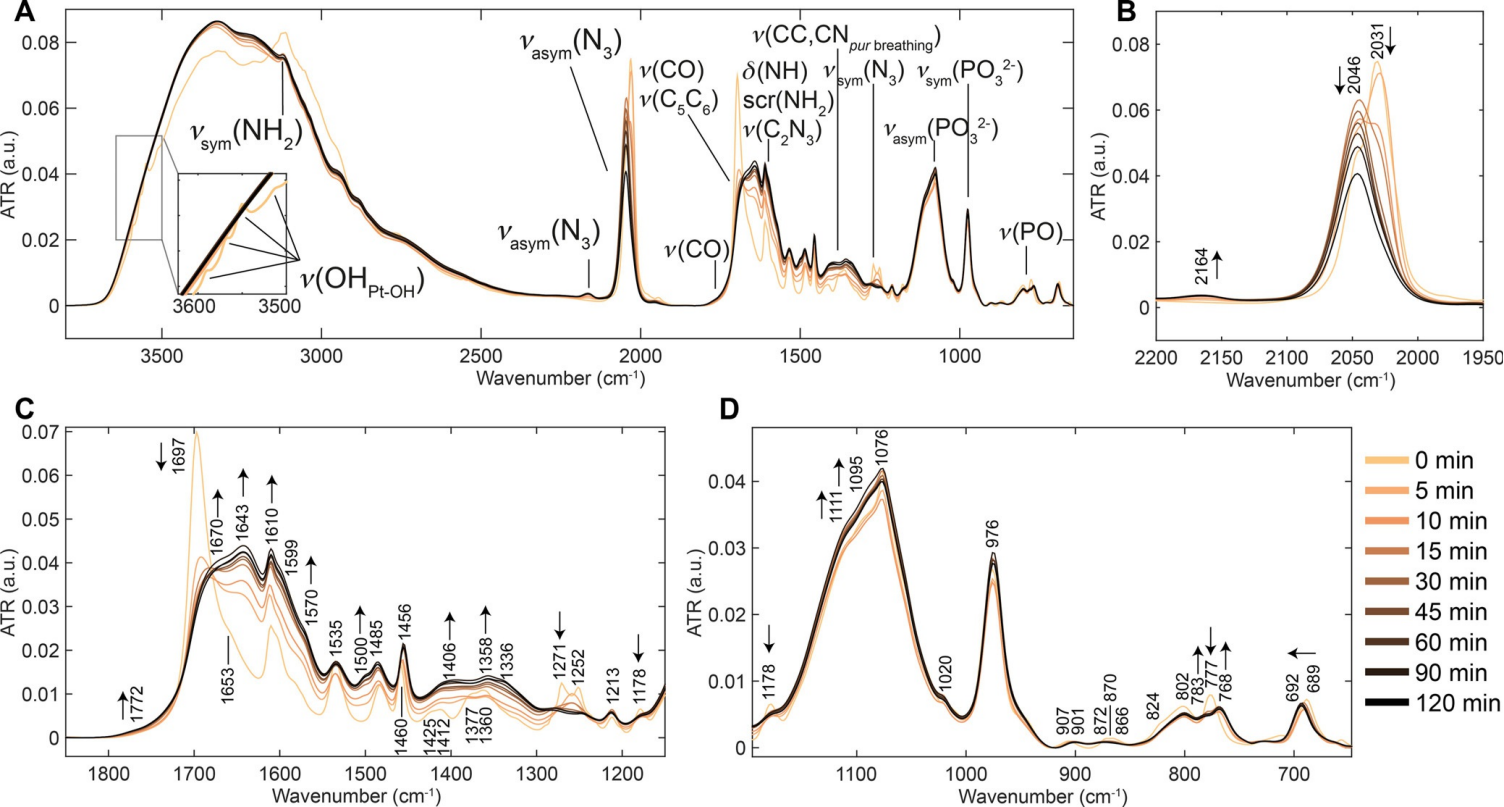
ATR‐FTIR spectra of the photodecomposition of **1** (2 mm)+5′‐GMP (2 mol equiv) under 420 nm irradiation. A) Full spectral range, 3800–645 cm^−1^, including main peak assignments. Refer to Figure S20 in the Supporting Information for a full description of peak vibrations. B) ATR‐FTIR spectra, 2200–1950 cm^−1^, highlighting the decrease and shifts in *ν*_asym_(N_3_) at 2031 cm^−1^ to 2046 cm^−1^ and the appearance of a new peak at 2164 cm^−1^. C) ATR‐FTIR spectra, 1850–1150 cm^−1^, highlighting the decrease in *ν*(CO) at 1697 cm^−1^, *ν*_sym_(N_3_) at 1271 cm^−1^ and *ν*(CC,CN_pyr breathing_) at 1178 cm^−1^, as well as the increase of several (new) peaks at 1172, 1670, 1643, 1610, 1570, 1500, 1406 and 1358 cm^−1^. D) ATR‐FTIR spectra, 1190–640 cm^−1^, highlighting the increase of the broad ν_asym_(PO_3_^2−^) peak at 1076 cm^−1^ and the shift of *ν*(PO) from 777 cm^−1^ to 768 cm^−1^. Notations used: *ν*=stretch, *δ*=in‐plane angle bending, sym=symmetric, asym=antisymmetric, pur breathing=purine breathing mode and arrows indicate direction of change to the vibration throughout irradiation.

The full spectral range (3800–645 cm^−1^) of the photoinduced reaction by ATR‐FTIR is shown in Figure 6 A, which includes the main peak assignments. Complete removal of the four *ν*(OH_Pt‐OH_) peaks (3591 cm^−1^, 3568 cm^−1^, 3550 cm^−1^, and 3512 cm^−1^) is observed within 5 min, whereas the broad *ν*(OH) feature (≈3300 cm^−1^) increases throughout irradiation. The symmetric NH_2_ stretching vibration (*ν*_sym_(NH_2_)) at 3113 cm^−1^ reduces in intensity and does not shift. Similarly, the other weak CH stretching vibrations remain present underlying the *ν*(OH) (Figure S1). Figure 6 B shows the changes occurring to the *ν*_asym_(N_3_) of **1**, which shifts from 2031 cm^−1^ to a single 2046 cm^−1^ during irradiation. At the same time a small peak appears at 2164 cm^−1^, which matches the infrared frequency of sodium azide.^13^

#### Photoinduced reaction of 1 with 5′‐GMP (1800–1150 cm^−1^)

Figure 6 C highlights the changes occurring between 1800 cm^−1^ and 1150 cm^−1^, in which, after 5 min of irradiation, the intensity of the *ν*(CO)+*ν*(C5C6) vibration at 1696 cm^−1^ is reduced significantly whilst a new peak appears at 1643 cm^−1^. Continuing irradiation results in a further increase to the 1643 cm^−1^ band, with the band at 1696 cm^−1^ shifting to 1670 cm^−1^ and a new weak band arising at 1772 cm^−1^. The scr(NH_2_)+*δ*(NH) vibration at 1610 cm^−1^ increases over time, whilst the shouldering *ν*(C4C5)+*ν*(N1C2) vibration at 1588 cm^−1^ shifts to 1599 cm^−1^ and 1570 cm^−1^ after 5 min and continues to increase in intensity throughout irradiation. Additionally, a new peak at 1500 cm^−1^ develops during irradiation shouldering the 1485 cm^−1^ vibration: scr(NH_2_)−*δ*(NH)+*δ*(C8 H)+*ν*(N7C8)+*ν*(C4N9). The *δ*(py,19b) vibration at 1460 cm^−1^ of **1** shifts to 1456 cm^−1^ during irradiation, which is identical to the results observed for the photodecomposition of **1**.

Four vibrations are present between 1450 cm^−1^ and 1300 cm^−1^ prior to irradiation, namely, the *ν*(C2′C3′)+δ(CH_sugar_) vibration at 1425 cm^−1^ and 1412 cm^−1^ as well as the purine ring breathing modes of 5′‐GMP at 1377 cm^−1^ and 1360 cm^−1^. Upon irradiation, these peaks broaden and increase in intensity, with the main peaks situated at 1406 cm^−1^, 1358 cm^−1^ and 1336 cm^−1^.

Both the symmetric azido stretching vibration, *ν*_sym_(N_3_), at 1271 cm^−1^ and the purine ring breathing mode, *ν*(C1N2)+*ν*(N3C4)+*ν*(C4C5)+*ν*(C4N9)−*ν*(C5N9), at 1178 cm^−1^ are reduced in intensity throughout irradiation.

#### Photoinduced reaction of 1 with 5′‐GMP (1150–640 cm^−1^)

The last spectral window between 1190–640 cm^−1^ in Figure 6 D, shows the changes to the phosphate‐related vibrations throughout irradiation. The broad antisymmetric phosphate stretching vibration, *ν*_asym_(PO_3_^2−^), peaking at 1076 cm^−1^ (*δ*(py,9a)) increases slightly during irradiation whilst the 1111 cm^−1^ and the new peak at 1095 cm^−1^ increase more notably. The symmetric phosphate stretching vibration, *ν*_sym_(PO_3_^2−^), at 976 cm^−1^ shows a minor increase in intensity during irradiation. The *ν*(PO) vibrations at 802 cm^−1^ and 777 cm^−1^ change throughout irradiation, in which the 777 cm^−1^ peak decreases and two new peaks at 783 cm^−1^ and 768 cm^−1^ develop and increase in intensity. Lastly, a minor shift in the *γ*(py,11)+*δ*(N_3_) vibration of **1** from 689 cm^−1^ to 692 cm^−1^ is observed, which is in line with the photodecomposition results of **1**.

### HPLC and MS: photoinduced reaction of 1 with 5′‐GMP

**Figure 7:**
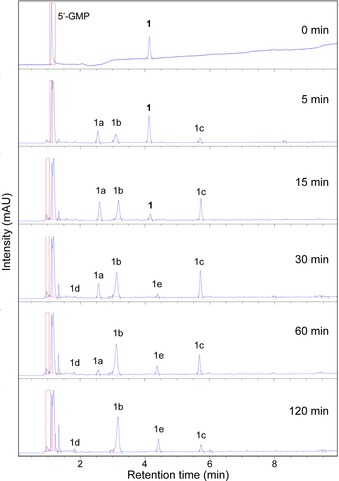
Analytical HPLC traces (254 nm) of the photoinduced binding of **1** (50 μm) with 5′‐GMP (1:2) upon 420 nm irradiation.

It is likely that several species are formed upon irradiation of **1** with 5′‐GMP, given the multistep photodecomposition of **1** and previously reported results.^4^ Monitoring the same reaction by analytical HPLC identified five potential photoproducts during 2 h of irradiation (Figure 7). Throughout the initial 15 min, three photoproducts (1a, 1b, and 1c) appear, while the signal intensity of **1** declines. Removal of **1** is reached after 30 min and two further photoproducts **(**1d and 1e) emerge. Further irradiation results in the reduction in intensity for 1a and 1c whilst 1b and 1e increase.

**Figure 8:**
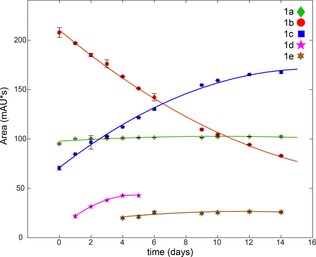
Evolution of HPLC peak areas of the photoproducts (1a–1e) after 420 nm irradiation of **1** (50 μm) and 5′‐GMP (1:2) for 30 min. Mean integrated peak areas of three measurements are shown for each photoproduct, trend lines were fitted using a 2nd order polynomial fit and standard deviations are depicted as error bars (black).

Separation of these photoproducts was carried out by preparative HPLC, as described in the Experimental Section. Figure S24 in the Supporting Information shows the HPLC retention times of the solution mixture with separated fractions matching the retention times of the mixture for photoproducts 1a, 1b, 1c, and 1e. Photoproduct 1d could not be separated by HPLC and was not investigated further. LCMS and HRMS signals were matched to possible species for 1a; [Pt(N_3_)(py)_2_(OH)_2_(5′‐GMP+2 H)]+Na^+^ (*m*/*z* 815.2, calcd *m*/*z* 815.1), 1b; *trans*‐[Pt(N_3_)(py)_2_(5′‐GMP‐2Na+H)+OH+H]^+^ (*m*/*z* 774.1355, calcd *m*/*z* 774.1113), 1c; *trans*‐[Pt(N_3_)(py)_2_(5′‐GMP‐2Na+2 H)]^+^ (*m*/*z* 758.1166, calcd *m*/*z* 758.1164) and 1e; *trans*‐[Pt(py)_2_(5′‐GMP+2H)_2_]^2+^ (*m*/*z* 539.6, calcd *m*/*z* 539.6) (Figure S24 and S25). Lyophilisation resulted in powders coloured yellow (1a), pale yellow/white (1b) and white (1c and 1e). The majority of separated product consisted of 1c (≈1 mg per photoreaction), followed by smaller quantities (<1 mg) for 1b, 1e, and 1a. Furthermore, the evolution of photoproducts in the dark, after 30 min of irradiation at 420 nm of **1** with 5′‐GMP, was monitored by analytical HPLC. Figure 8 shows the evolution of the HPLC peak areas of the photoproducts with matching retention times as previously labelled products (Figure 7). Mean areas and standard deviations are tabulated in Table S2. Photoproduct 1a increases slightly during the initial 24 h before stabilizing. Over the course of 14 days, 1b decreases while 1c increases. The presence of 1d is seen after 1 day until day 5, whereas 1e is first observed after 4 days and stabilizes after 12 days.

### ATR‐FTIR: separated photoproducts

ATR‐FTIR spectra of 1a, 1c and 1e were acquired and are shown in Figure S26 including peak labels. The *trans*‐platinum pyridine scaffold in the resulting photoproducts is maintained during lyophilisation, confirmed by the presence of the pyridine vibrations and/or antisymmetric azido stretching vibrations, identical to **1**. The pyridine bending modes, *δ*(py,8a), *δ*(py,19b) and *δ*(py,9a) are retained in the ATR‐FTIR spectra of 1a, 1c and 1e at 1614/1612/1612 cm^−1^, 1458/1456/1458 cm^−1^ and 1078/1076/1078 cm^−1^, respectively. Both 1a and 1c have a *ν*_asym_(N_3_) stretching vibration at 2044 and 2048 cm^−1^ correspondingly, whereas 1e shows a minor azido impurity at 2044 cm^−1^ (Figure S26). The formation of a weak band at 1772 cm^−1^ observed during the irradiation process (Figure 6) is observed in the ATR‐FTIR spectra of 1e at 1774 cm^−1^ (Figure S26). Similarly, the shifts observed by ATR‐FTIR throughout the irradiation of **1** with 5′‐GMP to the purine ring vibrations (CO, CC and CN stretching vibrations and NH/NH_2_ bending modes) are reflected in the spectra of 1c and 1e (Figure S26). The phosphate vibration, *ν*_asym_(PO_3_^2−^), of the separated photoproducts compared to the reaction mixture is shifted to higher frequencies, from 1076 cm^−1^ to 1136 cm^−1^ (1a, 1c) and 1132 cm^−1^ (1e), whereas the *ν*_sym_(PO_3_^2−^) vibration is reduced in intensity for 1a, 1c and 1e.

## Discussion

Photochemotherapy is attractive for the treatment of cancer because it uses non‐toxic prodrugs, which are activated only in the region of tumours with spatially directed light, so minimizing toxic side‐effects on healthy tissue. Photoactive Pt^IV^ complexes such as **1** show promise, being active against cancer cells at low micromolar doses and active in vivo using visible light. Moreover, they kill cancer cells by unusual mechanisms. It is important to explore methods to elucidate the photochemistry and photo‐biochemistry of these complexes. Although NMR spectroscopy is often informative for solution studies, in the present case, it is difficult to monitor the azido and hydroxido ligands because resonances for quadrupolar ^14^N are broad and ^15^N resonances cannot be enhanced by polarization transfer because there are no coupled protons.^14^ The present studies show that vibrational spectroscopy can make a significant contribution in this area.

The photodecomposition of **1** under 420 nm irradiation is likely to involve binding and/or interaction with water (solvent) when no other competing binding sites are present. ATR‐FTIR revealed the formation of a new OH bending mode underlying the peaks 1381 cm^−1^ and 1360 cm^−1^ (Figure 2 A and D). This broad band is reasonably assigned to the OH bending mode of water bound to platinum (*δ*(OH${}_{\mathrm{Pt}\text{-}\mathrm{OH}/\mathrm{OH}{}_{2}}$ )), behaving similarly to the *ν*(OH) and *δ*(OH) bands of water when exposed to a N_2_ flow during spectral acquisition (Figure S4). This is further supported by previous reports on platinum group metal hydroxides, where broad metal–OH bending modes were observed between 1600–1000 cm^−1^.^15^ Additionally, when 1 scan (≈1 sec) ATR‐FTIR measurements are taken continuously whilst turning the N_2_ flow ON and OFF, with two second intervals, the spectra change from one to another without intermediates visible (Figure S4), that is, the photodecomposed product of **1** rapidly takes up water vapour from air. This could suggest that free water is present in the final product matrix undergoing hydrogen bonding with Pt‐OH/OH_2_.

The PCA on the full ATR‐FTIR spectral region of the photodecomposition of **1** under 420 nm irradiation captures and correlates the effect of the removal of *ν*(OH_Pt‐OH_) at 3550 cm^−1^ with a decrease of −10 cm^−1^ to *ν*_asym_(N_3_) prior to an increase to *ν*_asym_(N_3_) of 21 cm^−1^ when little‐to‐no *ν*(OH_Pt‐OH_) is present. Furthermore, the appearance of the broad *δ*(OH${}_{\mathrm{Pt}\text{-}\mathrm{OH}/\mathrm{OH}{}_{2}}$ ) bands (1381 and 1334 cm^−1^) correlates to the *ν*_asym_(N_3_) at 2044 cm^−1^, where *δ*(OH${}_{\mathrm{Pt}\text{-}\mathrm{OH}/\mathrm{OH}{}_{2}}$ ) increases in intensity until the single *ν*_asym_(N_3_) vibration at 2044 cm^−1^ is completely removed as shown in Figure 2 A. Further deconstruction of the ATR‐FTIR spectra and investigation of the same reaction of **1** and its synthetic precursor **2** by UV/Vis and analytical HPLC were performed in order to connect these observed changes to possible formed intermediates.

Analytical HPLC revealed two possible intermediates with different retention times, which evolve at the same rate (P1 and P2) until one final product (P3) remains (Figure S6, S7 in the Supporting Information). Further, the steady state UV/Vis results and their MCR‐ALS spectra of **1** and **2** suggest that both compounds undergo photodecomposition through a similar intermediate before obtaining the final photoproduct, via a two‐step process (Figure S15, S16).

The deconstruction of the ATR‐FTIR spectra by MCR‐ALS, using the same kinetic model, revealed an intermediate (P1+P2) containing a single *ν*_asym_(N_3_) vibration at 2042 cm^−1^, the *δ*(OH${}_{\mathrm{Pt}\text{-}\mathrm{OH}/\mathrm{OH}{}_{2}}$ ) bands at 1379 cm^−1^ and 1334 cm^−1^ and all corresponding *trans* pyridine modes (Figure 2 A, S16), closely matching the PCA results.

This allows us to make a reasonable assignment for P1+P2 as *trans*‐[Pt^II^(N_3_)(py)_2_(H_2_O/OH)]. The MCR‐ALS spectrum of P3 is a close match to the final time points of the photodecomposition with no presence of azido vibrations observed, and can be reasonably assigned as *trans*‐[Pt^II^(py)_2_(H_2_O/OH)_2_] (Figure S17). Although there are some reports of stable Pt^III^ complexes, the general observed trend for Pt^IV^ reduction is the two‐photon reduction of Pt^IV^ to Pt^II^, which supports our findings.3c, 16

The formation of an intermediate species with a single azido ligand, *trans*‐[Pt(N_3_)(py)_2_(H_2_O/OH)] implies that reduction of **1** from Pt^IV^ to Pt^II^ occurs via release of at least one hydroxyl radical and one azidyl radical or two hydroxyl radicals. This is in line with the previously reported detection of azidyl radicals released from **1** upon irradiation by using spin traps.5b Recombination of two hydroxyl radicals results in formation of H_2_O_2_, which further decomposes to generate O_2_. The evolution of oxygen from the reduction of Pt^IV^ via hydroxyl radical release rather than from the solvent, has been reported to occur for a similar structure to **1**.^17^ Furthermore, the PCA of the ATR‐FTIR data suggest a possible second intermediate product during the first few minutes of irradiation, in which a −10 cm^−1^ reduction in wavenumber for *ν*_asym_(N_3_) is observed which correlates to the changing OH environment (removal of *ν*(OH_Pt‐OH_), Figure 3.

**Figure 9:**
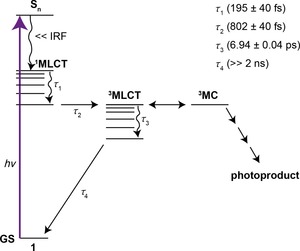
Proposed mechanism for the ultrafast photochemical processes of **1** and the extracted time‐constants (*τ*_n_) from the TEAS studies as discussed in the main text. IRF=instrument response function.

From the TEAS measurements and resulting global analysis, insight into the ultrafast photodynamics involved in photodecomposition of **1** is obtained. A possible photodecomposition mechanism is shown in Figure 9. However, further work is required to validate this scheme (e.g., transient vibrational spectroscopy) to assign intermediate states and high‐level electronic structure calculations (both beyond the scope of the present study). The initial excitation populates an array of singlet excited states, which undergo rapid internal conversion (IC), populating the lowest ^1^MLCT within our instrument response (≈80 fs). The excess vibrational energy imparted to this state undergoes intramolecular vibrational redistribution (IVR) and occurs with a time‐constant of *τ*_1_, as the EADS (*τ*_1_) is evidently broader and red‐shifted compared to EADS (*τ*_2_). This proceeds via intersystem crossing (ISC) to a vibrationally hot triplet excited state (*τ*_2_, ^3^MLCT). This conclusion is drawn from the evident change in spectral profile between EADS (*τ*_2_) and EADS (*τ*_3_), a sign of a state change. The timescale for this process also sensibly compares with that reported for other platinum complexes.^18^ The population of the vibrationally hot ^3^MLCT is likely capable of accessing an additional near degenerate state ^3^MC state. This near degeneracy in the two states might allow the population to flow freely between these states. Upon population of the ^3^MC state, the molecule can undergo rapid loss of one or more ligands, leading to the formation of the photoproduct. Our TEAS setup is blind to the formation of the photoproduct, as it absorbs beyond of the spectral window of our white light (<345 nm, see Figure S13). As the excess vibrational energy is shed to the solvent bath, access to the ^3^MC state is no longer available, switching off the formation of the photoproduct, with the remaining population becoming trapped in the vibrationally‐cold ^3^MLCT state. Furthermore, the emission spectrum of **1** reveals a band at ≈550 nm in acetonitrile upon 310 nm excitation (Figure S19). This large red‐shift points to either fluorescence, following a large geometry change in the excited state or, more likely, phosphorescence. The latter supports the idea of population becoming trapped in a triplet state. This vibrational cooling process and thus the loss of access to the ^3^MC state, occurs with a time‐constant of *τ*_3_, as the spectral features in EADS (*τ*_4_) are spectrally sharper and blue‐shifted compared to EADS (*τ*_3_). We note that our fitting procedure is unidirectional in population flow and does not include branched kinetics, therefore the fit convolutes the processes mentioned above into the time‐constant *τ*_3_. The vibrationally‐cold ^3^MLCT state persists beyond our maximum available Δ*t*.

Prior to this work, only theoretical studies had been carried out in order to investigate the ultrafast photodynamics of **1**. Analysis of Mulliken and NBO charges of the S_0_ and ^3^MLCT geometries of **1** revealed a loss in negative charge from the azido ligands and positive charge of platinum, including the elongation of platinum to nitrogen (azide) bond lengths, suggesting reductive elimination of the azides.^4^

Earlier computational work on a similar complex, *cis,trans,cis*‐[Pt(N_3_)_2_(OH)_2_(NH_3_)_2_], presented the accessibility of several photorelease pathways via low‐lying singlet states resulting in the release of the azide anion and low‐lying triplet states for which dissociation of ammonia and an azide anion was suggested without the reduction of platinum.8a Furthermore, Sokolov et al. carried out extended DFT screening of *cis,trans,cis*‐[Pt(N_3_)_2_(OH)_2_(NH_3_)_2_], *trans,trans,trans*‐[Pt(N_3_)_2_(OH)_2_(NH_3_)_2_] and a range of complexes of the formula *cis,trans,cis*‐[Pt(N_3_)_2_(OH)_2_(L)_2_] for their ground and excited state properties.8b The further inspection of their three lowest singlet excited states using the time‐dependent DFT method revealed the potential dissociative behaviour of the two azido ligands and photoreduction of platinum, from Pt^IV^ to Pt^II^. More recently, Shushakov et al. investigated the photophysical and photochemical processes of *cis,trans,cis*‐[Pt(N_3_)_2_(OH)_2_(NH_3_)_2_] and *trans,trans,trans*‐[Pt(N_3_)_2_(OH)_2_(NH_3_)_2_] on ps and μs timescales, capturing multistage photolysis for both complexes including initial substitution of one azide by a solvent molecule.^19^ Overall, the present findings by TEAS open up a new path for more comprehensive ultrafast photodynamic and high‐level theory studies of **1**, which are outside the scope of the present study.

The photoinduced reaction of **1** with 5′‐GMP was closely followed by ATR‐FTIR as shown in Figure 6. Prior to irradiation, splitting of the *ν*(OH) into four peaks is observed. This is indicative of increased hydrogen bonding between the hydroxyl groups of **1** and 5′‐GMP and/or water. Whilst 20 min of irradiation is required to completely remove the *ν*(OH) of **1** in water, the presence of 2 mol equiv of 5′‐GMP increases the rate of removal. The change in the antisymmetric azido vibration of **1** is in line with the photodecomposition of **1** in which after 2 h of irradiation, a single peak at 2046 cm^−1^ is present. Interestingly, a minor band emerges at 2164 cm^−1^ upon irradiation, which matches the infrared frequency of sodium azide. This suggests that the mechanism involves the release of the azide anion, N_3_^−^ from **1** in the presence of 5′‐GMP.

Large changes are observed to the purine‐ring‐related CO, CC, and CN stretching vibrations and NH/NH_2_ bending modes of 5′‐GMP in the 1800–1485 cm^−1^ ATR‐FTIR region throughout irradiation. Firstly, a reduction in intensity of the *ν*(CO)+*ν*(C5C6) vibration at 1696 cm^−1^ is observed. Similar results were obtained for several M^2+^–5′‐GMP complexes by Theophanides and co‐workers, in which a similar reduction in intensity of this band is observed but only small changes to the wavenumber itself (≈5 cm^−1^).^20^ This was attributed to the maintained hydrogen bonding between C6O of 5′‐GMP and water bound to the metals as observed in the crystal structures. In this case a clear shift is observed for the *ν*(CO)+*ν*(C5C6) to 1670 cm^−1^. This could be due to the pyridines of **1** shielding the C6O from undergoing hydrogen bonding. The DFT optimized S_0_ structures of potential photoproducts, *trans*‐[Pt(N_3_)(py)_2_(5′‐GMP)]^1−^ and *trans*‐[Pt(py)_2_(5′‐GMP)_2_]^2−^, show a 2.333 Å, 2.250 Å and 2.293 Å distance between the C6O oxygen and a pyridine proton (Figure S20). Furthermore, the DFT‐predicted frequencies for this vibration shift from 1705 cm^−1^ to 1681 cm^−1^, going from free 5′‐GMP to **1** bound to the N7 position of 5′‐GMP (*trans*‐[Pt(N_3_)(py)_2_(5′‐GMP)]^1−^) (Table S1). In the case of *trans*‐[Pt(py)_2_(5′‐GMP)_2_]^2−^, two ν(CO)+ν(C5C6) peaks are predicted at 1687 cm^−1^ and 1682 cm^−1^. These predicted frequencies decreasing by 25/18/23 cm^−1^ are well matched by the experimental value of 27 cm^−1^. Stronger infrared absorption is observed for the NH/NH_2_ bending modes at 1610 cm^−1^, which indicates the decrease in hydrogen bonding. Continued increase of the new *ν*(C4C5)+*ν*(N1C2) vibrations at 1599 cm^−1^ and 1570 cm^−1^ are further indications of binding of **1** to 5′‐GMP. The changes occurring to the breathing modes of 5′‐GMP (from 1377/1360 cm^−1^ to 1358/1336 cm^−1^) are likely to be influenced by the binding of platinum to the N7 position of 5′‐GMP, lowering the infrared wavenumber value for this vibration. This can be attributed to changes to the hydrogen bonding network upon platination of the N7 position of the pyrimidine ring system.^20^, ^21^ The earlier assigned *δ*(OH${}_{\mathrm{Pt}\text{-}\mathrm{OH}/\mathrm{OH}{}_{2}}$ ) bands during the photodecomposition of **1** in water at 1381 cm^−1^ and 1334 cm^−1^ are in close proximity to the breathing modes of 5′‐GMP, thus the presence of water bound platinum vibrations cannot be ruled out. Interestingly, the decrease in intensity of the purine ring breathing mode at 1178 cm^−1^ throughout irradiation is predicted by the DFT calculations, in which a ≈fivefold decrease in intensity is observed when platinum binds to N7 of 5′‐GMP. Perturbation of the electronic structure upon binding to the purine ring could be the cause of the observed decrease in intensity. Earlier reported direct binding of phosphate to magnesium resulted in a shift of +18 cm^−1^ to the *ν*_sym_(PO_3_^2−^) vibration, whereas indirect interaction of the water bridging between phosphate and copper or magnesium resulted in a split in this vibration into two peaks.^20^, ^21^ Reported platinum 5′‐GMP complexes showed less effect on the phosphate‐related stretching vibrations, in line with our results, which were reported to be primarily due to conformational changes around the phosphate and sugar moiety.^20^, ^21^ Thus, binding of the phosphate to **1** might be ruled out. In general, broadening of vibrations at and near 3300 cm^−1^ and 1650 cm^−1^ is observed throughout the photoinduced reaction of **1** and 5′‐GMP, which indicate the presence of water in the resulting product matrix with *ν*(OH) and *δ*(OH) underlying other vibrations, respectively (Figure 6).

The likelihood of several photoproducts arising from irradiation of **1** in the presence of 5′‐GMP was confirmed by analytical HPLC. Five potential photoproducts were identified of which four were separated by preparative HPLC and assigned by MS. These include [Pt(N_3_)(py)_2_(OH)_2_(5′‐GMP)] (1a), *trans*‐[Pt(N_3_)(py)_2_(5′‐GMP)+OH+H]^+^ (1b), *trans*‐[Pt(N_3_)(py)_2_(5′‐GMP)]^+^ (1c) and *trans*‐[Pt(py)_2_(5′‐GMP+2H)_2_]^2+^ (1e) of which the *trans* pyridine scaffolds were retained as shown by the ATR‐FTIR spectra (Figure S26). Previous studies showed the release of azidyl radicals from **1** on its own in water, with introduction of 5′‐GMP hardly affecting azidyl radical formation.5b Photoproduct 1e is a minor product; this might suggest that the small azide band observed during the irradiation of **1** and 5′‐GMP (Figure 6) is due to the release of the second azido ligand from **1** as an azide anion.

The ATR‐FTIR spectra of separated photoproducts correlate well to the observed changes throughout irradiation of **1** with 5′‐GMP, especially in the 1800–1300 cm^−1^ region. Larger changes between the ATR‐FTIR spectra of the mixture and separated photoproducts were seen in the phosphate region. The separation by HPLC and lyophilisation is expected to change the chemical environment and molecular geometry around PO_3_^2−^, the charge depends on pH where counter ions might be exchanged. This could account for the observed increase in wavenumber values where fewer short distance interactions are present compared to the reaction mixture containing 2 mol equiv of the disodium 5′‐GMP salt.

Photoproduct 1b decomposed after lyophilisation according to MS and no ATR‐FTIR spectra could be obtained. HRMS results prior to lyophilisation match a Pt^IV^ species (*trans*‐[Pt^IV^(N_3_)(OH)(py)_2_(5′‐GMP‐2Na)]+H^+^). Initial studies of **1** revealed the formation of a minor Pt^IV^ species upon irradiation in presence of 5′‐GMP.^4^ Further studies of derivatives of **1** revealed the oxidizing capabilities of such complexes, where oxidation on the C8 position of 5′‐GMP was postulated.^17^ The *m*/*z* of a Pt^II^ species bound to an oxidized 5′‐GMP (*trans*‐[Pt^II^(N_3_)(py)_2_(5′‐GMP‐2Na‐H+OH)]^1−^+2H^+^) is identical to the proposed Pt^IV^ species and therefore cannot be ruled out.

The evolution of species 1a to 1e in the dark after 30 min of irradiation captured changes to absorption intensity of each photoproduct during the course of two weeks. The decrease in absorption intensity of 1b could be due to the reduction of the proposed Pt^IV^ species to yield the Pt^II^ species 1c, which increases in absorption intensity over time.

## Conclusions

This work has demonstrated the potential for ATR‐FTIR to elucidate the photodecomposition of the photoactivatable diazido Pt^IV^ anticancer prodrug candidate (**1**) including the photoinduced reaction of **1** with 5′‐GMP. Principal Component Analysis (PCA) captured and correlated the observed changes of the disappearance of *ν*(OH_Pt‐OH_) at 3550 cm^−1^ with a decrease of −10 cm^−1^ to *ν*_asym_(N_3_) prior an increase to *ν*_asym_(N_3_) of 21 cm^−1^ when little to no ν(OH_Pt‐OH_) is present. Furthermore, the appearance of a new broad *δ*(OH${}_{\mathrm{Pt}\text{-}\mathrm{OH}/\mathrm{OH}{}_{2}}$ ) band (1381 cm^−1^ and 1334 cm^−1^) correlates to the single *ν*_asym_(N_3_) vibration at 2044 cm^−1^, where *δ*(OH${}_{\mathrm{Pt}\text{-}\mathrm{OH}/\mathrm{OH}{}_{2}}$ ) gradually increased until the single *ν*_asym_(N_3_) at 2044 cm^−1^ completely disappeared.

PCA and extended experimental studies indicated the formation of *trans*‐[Pt(N_3_)(py)_2_(OH/H_2_O)] as an intermediate throughout 420 nm irradiation of **1** in water by HPLC and Multi‐Curve Resolution Alternating Least Squares (MCR‐ALS) of the ATR‐FTIR and UV/Vis spectroscopy, with the acetonitrile studies (310 nm excitation) following the same two‐step pathway. Complete removal of the last azide required extended irradiation times to reach the final product *trans*‐[Pt(py)_2_(OH/H_2_O)_2_].

Transient Electronic Absorption Spectroscopy (TEAS) provide a probable vibrational switching mechanism for **1** where the excited state complex has access to the dissociative ^3^MC state only when it is vibrationally hot, after which it remains trapped in a vibrationally cool state with a long‐lived absorption (≫2 ns). This paves the way for future comprehensive ultrafast photodynamic (transient vibrational absorption spectroscopy) and high‐level theory studies of **1**, which were outside the scope of this study.

The ATR‐FTIR of the photoinduced reaction of **1** with 5′‐GMP exposed the faster removal of *ν*(OH) compared to the photodecomposition (≤5 min vs. 20 min), whilst showing increased hydrogen bonding between the hydroxyl groups of **1** and 5′‐GMP and/or water prior to irradiation. Changes to the antisymmetric azido vibration are in line with the photodecomposition results with only a single vibration present after two hours of irradiation and additionally a new vibration matching sodium azide, that is, release of an azide anion. Comprehensive ATR‐FTIR assignments of 5′‐GMP and photoproducts formed upon irradiation of **1** and 5′‐GMP were achieved by DFT calculations. Separation of photoproducts by HPLC, followed by MS and ATR‐FTIR allowed a comprehensive ATR‐FTIR assignment. These further aided the detailed elucidation of the observed changes in the ATR‐FTIR of the reaction mixture throughout irradiation. For instance, *trans*‐[Pt(N_3_)(py)_2_(5′‐GMP)] is the major product in the reaction mixture, however a marker band for the *trans*‐[Pt(py)_2_(5′‐GMP)_2_] product at 1772 cm^−1^ allows the tracking of this product.

These studies show that vibrational spectroscopy can make a major contribution to understanding the chemical basis for the mechanism of action of photoactivatable diazido Pt^IV^ prodrugs including their interactions with DNA bases.

## Experimental Section

### Materials

All reactions were performed under nitrogen atmospheres using standard Schlenk techniques. K_2_PtCl_4_ (99 %) was purchased from Precious Metals Online. Guanosine 5′‐monophosphate disodium salt hydrate ≥99 % (5′‐GMP) and all other chemicals and solvents were purchased from Sigma–Aldrich and used as received.

### Instrumentation and methods

UV/Vis spectra were acquired on a Varian Cary 300 BIO UV/Vis spectrophotometer equipped with a Varian Cary temperature controller (298 K, unless otherwise stated).

LCMS spectra were acquired with an Agilent 6100 Series Single Quadrupole LC/MS incorporating a photodiode array detector (214/254 nm) coupled directly to an electrospray ionization source.

HRMS was conducted using an Agilent 6224 TOF LC/MS Mass Spectrometer coupled to an Agilent 1290 Infinity. All data were acquired and reference‐mass corrected via a dual‐ESI source.

Fluorescence spectra were recorded on a Horiba Fluorolog 3 spectrophotometer using an excitation wavelength of 310 nm with a bandwidth of 8 nm. The data obtained for **1** were only usable between 363 and 580 nm due to scatter and 2nd order effects.

High‐performance liquid chromatography was carried out on a Agilent 1260 Infinity Analytical HPLC incorporated with an Infinity diode array detector (214/254) with a Agilent Zorbax Eclipse Plus C‐18 Rapid Resolution column (95 Å, 3.5 μm, 100×4.6 mm). All samples were run using eluent A: H_2_O (0.1 % trifluoroacetic acid, TFA), B: acetonitrile (0.1 % TFA), flow: 1 mL min^−1^, 5–25 % B varying depending on sample. Semi‐preparative HPLC was performed on an Agilent 1260 Infinity Preparative‐scale Purification system incorporated with an Infinity diode array detector with a Agilent Zorbax SB‐C18 column (80 Å, 5 μm, 150×9.4 mm). Conditions; A: H_2_O (0.1 % TFA), B: acetonitrile (0.1 % TFA), flow: 5 mL min^−1^, 0–20 min: 2–8 % B, 20–60 min: 8–25 % B, 60–63 min: 25–100 % B).

A Rayonet RPR‐200 photoreactor equipped with six lamps was used for 420 nm irradiation (*λ*_em max_: 420 nm, spectral distribution: 390–460 nm, ≈3.6 mW cm^−2^). Samples were irradiated in a 3 mL, 1 cm pathlength fluorescent quartz cuvette with a PTFE stopper placed in the centre of the photoreactor. A Tunable KiloArc™ Illuminator equipped with a 1000 W Xenon lamp was used for 310 nm irradiation (*λ*_em max_: 310 nm, spectral distribution: 290–320 nm, ≈8.0 mW). Samples were irradiated in an 800 μL, 1 cm pathlength quartz cuvette sealed with a PTFE stopper.

ATR‐FTIR spectra were acquired on a Bruker model Equinox 55 FT‐IR spectrometer with a N_2_‐cooled mercury‐cadmium‐telluride (MCT) detector and a Harrick silicon multiple reflection ATR accessory. OPUS software 6.0 was used to acquire ATR spectra (wavenumber range: 4000‐400 cm^−1^, spectral resolution: 4 cm^−1^, and 50 interferograms co‐added). 3 μL samples were deposited on the ATR, evaporated under a gentle N_2_ flow (typically 1–3 min) and the resulting thin films were measured accordingly.

### Transient electronic absorption spectroscopy (TEAS)

The experimental procedures pertaining to TEAS have been described in detail elsewhere and a brief overview is presented herein.^22^ The femtosecond pump pulses were generated using a commercially available optical parametric amplifier, (TOPAS‐C, Spectra‐Physics). The white light continuum (330–690 nm) utilized as the probe pulse was produced through supercontinuum generation from the 800 nm fundamental in a 2 mm thick CaF_2_ window; translated vertically. The pump pulse wavelength was set to 310 nm (4.00 eV). The fluence of the pump beam was set between 1–2 mJ cm^−2^. The difference between the pump and probe polarizations was held at magic angle (54.7°) to negate rotational effects using a half‐wave plate in the probe beam path. The pump‐probe time delay (Δ*t*) was varied by adjusting the optical delay of the probe pulse, the maximum obtainable Δ*t* was 2 ns. Changes in the optical density (ΔOD) of the samples were calculated from probe intensities, collected using a spectrometer (Avantes, AvaSpec‐ULS1650F). Samples of **1** were made to a concentration of 2 mm in acetonitrile (99.9 %, VWR). The delivery system for the samples was a flow‐through cell (Demountable Liquid Cell by Harrick Scientific Products Inc.) with a 100 μm path. The sample was circulated using a peristaltic pump (Masterflex) recirculating sample from a reservoir to provide each pulse with fresh sample.

### Theoretical calculations

DFT calculations were carried out using the Gaussian 9 package^23^ with the hybrid PBE0 functional with 25 % HF exchange.^24^ cc‐pVDZ was used for carbon, hydrogen, nitrogen, oxygen and phosphorus, whereas for platinum an augmented cc‐pVDZ‐PP with effective core potential (ECP) was used.^25^ The conductor‐like polarizable continuum model (CPCM) was applied (water) to better describe the electrostatic interactions between metal to ligand bonds.^26^ Calculations converged to optimised geometries by allowing all parameters to relax, corresponding to true energy minima as confirmed by the lack of imaginary frequencies. The ground‐state geometries reported previously for **1** were used as a starting guess and 5′‐GMP starting geometries were obtained using universal force field optimizations, prior to a full conformational screening by systematic variation of all the dihedral angles until corresponding minima were attained.^6^ Sodium ions did not influence the geometry optimization of 5′‐GMP and were therefore omitted. Starting geometry estimates for photoproducts of **1** and 5′‐GMP were based on the S_0_ geometries of each, respectively, with conformational screening carried out by systematically rotating along the platinum–N7 axis and varying the dihedral angles between platinum and the N7 position of 5′‐GMP until consequent minima were reached. Two S_0_ geometries for *trans*‐[Pt(py)_2_(5′‐GMP)_2_]^2−^ and one for *trans*‐[Pt(N_3_)(py)_2_(5′‐GMP)]^1−^ were obtained (Figure S20). Calculated infrared spectra were exported from GaussView 5 with a half‐width of 16 cm^−1^.

### Data processing

PLS_Toolbox 8.2 (Eigenvector Research) was used to carry out the Principal Component Analysis (PCA). ATR‐FTIR spectra of the photodecomposition of **1** were cut (3700‐640 cm^−1^), Standard Normal Variate (SNV) scaled, mean centred and cross‐validated (venetian blinds, 10 splits and 1 sample per split). Three principal components (PC) were selected, quantifying the majority of changes in the spectra over time.

OPUS 7.2 (Bruker Optics) software was used to carry out the pre‐processing of ATR‐FTIR data for all other processes. ATR‐FTIR spectra were cut (3800–640 cm^−1^ for **1** and 3800–645 cm^−1^ for **1**+5′‐GMP), vector normalized (to the band at 1625–1595 cm^−1^ for **1** and 3800–645 cm^−1^ for **1** + 5′‐GMP), rubberband baseline corrected and replicates were averaged.

The Multi‐Curve Resolution Alternating Least Squares (MCR‐ALS) toolbox 2.0 for Matlab was used to carry out the curve fitting for the ATR‐FTIR and UV/Vis spectra.^27^ Component estimates were obtained through the inbuilt singular value decomposition algorithm, where the initial components where set to those of eigenvalues ≥1 and the observed species by analytical HPLC. The final component numbers were verified via variation of the time point inputs, component number and applying kinetic model constraints. Initial pure spectra estimates were determined through the purest variable detection.^27^ Non‐negative least squares (nnls) was set to apply non‐negativity for components concentrations and pure spectra. Kinetic constraints were set to follow the generic scheme: A>P1>Px with rate constants *k*_x_ (A=**1** or **2**). Concentrations of A at time 0 were set to the corresponding concentrations in the experiment, that is, 50 μm for UV/Vis and 2 mm for ATR‐FTIR. The convergence limit was set to 1×10^−3^ and this criteria was typically met within 50 iterations.

### Photodecomposition of 1 in water

Solutions (2 mL) of **1** in Mili‐Q water (2 mm) were irradiated in a 1 cm pathlength quartz fluorescence cuvette. 30 μL aliquots were taken from the solution during irradiation at selected time points until completion of photodestruction (every minute between 0–15 min, 20, 25, 30, 45 min and 1, 1.5, 2, 2.5, 3.5, 8 and 22 h). Each aliquot was stored in the dark until measurement by ATR‐FTIR, Analytical HPLC and UV/Vis. The photodecomposition at 2 mm concentrations were carried out in triplicate, with each time point measured twice by ATR‐FTIR, once by Analytical HPLC and only selected time points by UV/Vis, which were compared to the photodecomposition of a 50 μm solution of **1**. Samples for Analytical HPLC and UV/Vis were diluted to 50 μm prior to measurement. The photodecomposition of **1** at 50 μm concentrations by UV/Vis (3 mL) was carried out in triplicate until completion (every minute between 0–15 minutes, 20, 23, 30, 45, 60, 90, 120 and 150 min). The stability of **1** and its aliquots after irradiation in the dark at 310 K was monitored by UV/Vis for 64 h and no changes were observed (Figure S10). The photodecomposition of **1** at 50 μm and 2 mm concentrations under 420 nm irradiation follow the same evolution where only the rate differs (Figure S9).

### Photodecomposition of 1 and 2 in acetonitrile

The photodecomposition of **1** and **2** in acetonitrile (50 μm) under 310 nm irradiation monitored by UV/Vis (3 mL) in 1 cm pathlength quartz cuvettes was carried out in triplicates until completion.

### Photoinduced reaction of 1 with 5′‐GMP

Solutions (2 mL) of **1** (2 mm) and 5′‐GMP (4 mm) in Mili‐Q water were irradiated a 1 cm pathlength quartz fluorescence cuvette for 2 h. 50 μL aliquots were taken from the solution during irradiation at selected time points (0, 5, 10, 15, 30, 45, 60, 90 and 120 min). Samples were protected from light and stored on ice prior to measurement by ATR‐FTIR. The photoinduced reaction was carried out in triplicate, with each time point measured at least twice by ATR‐FTIR. A 2 mL solution of 5′‐GMP (4 mm) was exposed to 24 h of irradiation and measured by ATR‐FTIR. The stability of **1** (2 mm) and 5′‐GMP (2 mol equiv) was confirmed by ATR‐FTIR and HPLC for 35 days (Figure S22 and S23) and 22 h of irradiation of 5′‐GMP (4 mm) showed no changes by ATR‐FTIR (Figure S21).

### Separation of photoproducts formed by irradiation of 1 with 5′‐GMP

Solutions (3 mL) of **1** (3.58–3.86 mm) and 5′‐GMP (1:2) in Mili‐Q water were irradiated for one hour in a 1 cm pathlength quartz fluorescence cuvette prior to separation by semi‐preparative HPLC. Fractions were lyophilised and protected from exposure to light. Analytical HPLC was carried out before and after irradiation, after separation and after lyophilisation. Pure fractions (>95 % according to HPLC) were stored in the freezer and protected from light. Samples were dissolved in Mili‐Q water prior to ATR‐FTIR, LCMS and HRMS analysis.

### Evolution of photoproducts after irradiation of 1 with 5′‐GMP

A 1.5 mL solution of **1** and 5′‐GMP (50 μm **1**, 100 μm 5′‐GMP) in a glass LCMS vial was irradiated for 30 min. Analytical HPLC measurements were taken before irradiation and after irradiation, t: 0, 1, 2, 3, 4, 5, 6, 9, 10, 12 and 14 days. Analytical HPLC measurements were carried out in triplicate and peaks were auto integrated (slope sensitivity: 5 mAU s^−1^, peak width: 0.02 min, area reject: 5 mAU*s, height reject: 2 mAU, shoulders: off). Analytical HPLC samples were directly taken from the vial and punctured stoppers were replaced after each run. The vial was stored at RT and covered from light in between measurements.

### Synthesis

*trans,trans,trans*‐[Pt(N_3_)_2_(OH)_2_(py)_2_] **(1)** and *trans,trans,trans*‐[Pt(N_3_)_2_(py)_2_] (**2**) were synthesized as reported previously^6^ with characterization data in agreement with those reported originally.^4^ ***Caution**! Heavy metal azides are known shock sensitive detonators. Whilst no problems were encountered during this work, it is crucial not to apply excessive pressure to platinum azido compounds. Exposure of **1** and **2** to light was strictly limited during synthesis, sample preparation and measurements*.

## Conflict of interest

The authors declare no conflict of interest.

## Supporting information

As a service to our authors and readers, this journal provides supporting information supplied by the authors. Such materials are peer reviewed and may be re‐organized for online delivery, but are not copy‐edited or typeset. Technical support issues arising from supporting information (other than missing files) should be addressed to the authors.

Supplementary
