# Supplementary material for: Spectroscopic Studies on Photoinduced Reactions of the Anticancer Prodrug, trans,trans,trans‐[Pt(N3)2(OH)2(py)2]
Source: Chemistry. 2018 Feb 5;24(22):5790–803. doi: 10.1002/chem.201705349 (PMC5947305; doi:10.1002/chem.201705349)
Supplement: Supplementary file 1 — Supplementary [file CHEM-24-5790-s001.pdf]

CHEMISTRY
A European Journal

Supporting Information

Spectroscopic Studies on Photoinduced Reactions of the Anticancer Prodrug, *trans,trans,trans*-[Pt(N_3)$_2$(OH)$_2$(py)$_2$]

Robbin R. Vernooij,[a, b] Tanmaya Joshi,[c] Michael D. Horbury,[b] Bim Graham,[d] Ekaterina I. Izgorodina,[a] Vasilios G. Stavros,[b] Peter J. Sadler,*[b] Leone Spiccia†,[a] and Bayden R. Wood*[a]

chem_201705349_sm_miscellaneous_information.pdf

Contents

Comments on Experimental and Calculated Infrared Frequencies

Table S1. Main experimental and calculated infrared frequencies for the 5'-GMP moiety of 5'-GMP, *trans*-[Pt(N_3)($py)_2$(5'-GMP)]$^{1-}$ and *trans*-[Pt($py)_2$(5'-GMP$)_2$]$^{2-}$ (1 and 2).
Table S2. Mean HPLC peak areas and standard deviations of the photoproducts of **1** (50 μM) and 5'-GMP (100 μM) monitored after 30 min of 420 nm irradiation.

Figure S1. ATR-FTIR spectra including peak labels and assignments of **1**, **1** + 2 mol equiv. 5'-GMP and 5'-GMP.
Figure S2. Second derivative ATR-FTIR intensity of v(OH_{Pt-OH}) at 3550 cm^{-1} of **1** over time.
Figure S3. ATR-FTIR spectra of water (blue) and the photodecomposition of **1** (2 mM in water).
Figure S4. ATR-FTIR spectra of the final photoproduct of the photodecomposition of **1** (2 mM in water).
Figure S5. PCA analysis results for the photodecomposition of **1** in water under 420 nm irradiation by ATR-FTIR.
Figure S6. Analytical HPLC traces (254 nm) for the photodecomposition of **1** (2 mM in water) under 420 nm irradiation.
Figure S7. Analytical HPLC trace (254 nm) for **1** in water after 8 min of 420 nm irradiation.
Figure S8. UV-vis spectra for the photodecomposition of **1** (50 μM in water) under 420 nm irradiation.
Figure S9. UV-vis spectra of the photodecomposition of **1** in water under 420 nm irradiation.
Figure S10. UV-vis spectra of **1** after 10 min of 420 nm irradiation at 310 K and a further 64 h at 310 K.
Figure S11. UV-vis spectra of **1** in acetonitrile (50 μM, bottom and 2 mM, top).
Figure S12. UV-Vis spectra of **2** in acetonitrile.
Figure S13. UV-Vis spectra of **1** in acetonitrile (50 μM) upon 310 nm irradiation.
Figure S14. UV-Vis spectra of **2** in acetonitrile (50 μM) upon 310 nm irradiation.

Figure S15. UV-Vis spectra of **1** and **2** in acetonitrile upon 310 nm irradiation.

Figure S16. Deconstructed UV-vis spectra of the photodecomposition of **1** and **2** by MCR-ALS.

Figure S17. Deconstructed ATR-FTIR spectra of the photodecomposition of **1** by MCR-ALS.

Figure S18. TAS residual plot.

Figure S19. Emission spectrum of **1** in acetonitrile under 310 nm excitation.

Figure S20. Lowest energy geometries optimized by DFT for *trans*-$[Pt(N_3)(py)_2(5'\text{-}GMP)]^{1-}$, *trans*-$[Pt(py)_2(5'\text{-}GMP)_2]^{2-}$ and *trans*-$[Pt(py)_2(5'\text{-}GMP)_2]^{2-}$.

Figure S21. ATR-FTIR of 5'-GMP before (black) and after 22 hours (red) of 420 nm irradiation.

Figure S22. Analytical HPLC traces for reaction of **1** with 5'-GMP (1:2) in the dark at 0 min and after 35 d.

Figure S23. ATR-FTIR of **1** with 5'-GMP (1:2) in the dark immediately after mixing and after 35 d.

Figure S24**.** Analytical HPLC traces and MS data for the photoinduced reaction of **1** with 5'-GMP (1:2).

Figure S25. HRMS scans and *m/z* expansions of separated fractions formed during the irradiation of **1** with 5'-GMP (1:2) under 420 nm irradiation.

Figure S26. ATR-FTIR of **1** with 5'-GMP (1:2) under 420 nm irradiation and separated photoproducts.

Experimental and Calculated Infrared Frequencies for 5’-GMP, *trans*-[Pt(N_3)(py)$_2$(5’-GMP)]$^{1-}$ and *trans*-[Pt(py)$_2$(5’-GMP)$_2$]$^{2-}$.

A uniform scaling factor of 0.9496 was applied to the calculated infrared frequencies, based on the average scaling factors of three major experimental vibrations of 5’-GMP (1697 cm^{-1}, 1610 cm^{-1} and 1078 cm^{-1}) to best match the 1800 – 640 cm^{-1} region.

Table S1. Main experimental and calculated infrared frequencies for the 5’-GMP moiety in 5’-GMP, *trans*-[Pt(N_3)(py)$_2$(5’-GMP)]$^{1-}$ and *trans*-[Pt(py)$_2$(5’-GMP)$_2$]$^{2-}$ (**1** and **2**) including predominant vibrational modes.

Experimental (cm^{-1})[a]	Calculated (cm^{-1})[b]					Assignment[c]
5’-GMP	5’-GMP	5’-GMP	*trans*-[Pt(N_3)(py)$_2$(5’-GMP-2Na)]$^{1-}$	*trans*-[Pt(py)$_2$(5’-GMP-2Na)$_2$]$^{2-}$ (**1**)	*trans*-[Pt(py)$_2$(5’-GMP-2Na)$_2$]$^{2-}$ (**2**)	
	PBE0	CAM-B3LYP	PBE0	PBE0	PBE0	
3321 s, br	3535	3524	3546	3546<br>3542	3546<br>3542	$\nu_{asym}(NH_2)$
~3300 s, br	3386	3393	3497	3497	3497	ν(OH)
-	3428	3420	3428	3428<br>3427	3429<br>3426	ν(NH)
3113 s, br	3422	3414	3424			$\nu_{sym}(NH_2)$
3016 sh<br>-<br>2763 sh	2974<br>-<br>2847	2982<br>-<br>2860	2967<br>-<br>2863	2966<br>-<br>2870	2958<br>-<br>2859	$\nu(CH_{sugar})$
	2801	2801	2706	2703	2741	$\nu(C_8H)$
1697 s<br>1653 m, sh	1705	1705	1680	1687 (+)<br>1682 (-)	1701<br>1695	$\nu(C_1O) + \nu(C_5C_6) + \delta(NH) - scr(NH_2)$
1610 m	1584	1583	1586	1587 (+)<br>1588 (-)	1588 (+)<br>1587(-)	$scr(NH_2) + \delta(NH) + \nu(C_2N_3) - \nu(N_1C_6)$
1581 m, sh	1554	1558	1570	1570	1574<br>1572	$\nu(C_4C_5) + \nu(N_1C_2) + wag(NH_2) - \delta(NH)$
1535 m	1540	1541	1538	1539<br>1536	1540 (+)<br>1539 (-)	$scr(NH_2) + \nu(C_2N_3) + \nu(C_4N_9) + \nu(C_5C_6) + \nu(N_7C_8)$
1485 m	1510	1513	1505	1507<br>1501	1509 (+)<br>1507(-)	$scr(NH_2) - \delta(NH) + \delta(C_8H) + \nu(N_7C_8) + \nu(C_4N_9)$
1465 w, sh	1466	1469	1472	1476<br>1471	1483<br>1480	$\delta(C_8H) + \nu(N_7C_8) + \nu(C_4C_5) + \nu(N_1C_2) + \delta(NH)$
1419 w, sh<br>1414 w	1412	1418	1411	-	-	$\nu(C_{2'}C_{3'}) + \delta(CH_{sugar})$
-	1401	1404	1406	1409	1412	$\nu(N_7C_8) + \nu(C_4N_9) + \delta(C_8H) + \nu(C_4C_5)$
-	1365	1365	1385	1396<br>1384	1388<br>1379	$\delta(CH_2)$
1377 m, sh<br>1360 m	1358	1353	1347	1363<br>1353	1374<br>1354	$\nu(C_1C_5) + \nu(C_{1'}C_9) + \delta(C_8H) + \nu(N_{1'}C_2) + wag(NH_2)$ (ring breathing mode, nr 4-9)
-	1331	1336	1332	1335	1338	$\nu(C_8N_9) + \nu(C_5N_7) +$

					1335	$\delta(CH_{sugar})$
-	1309	1312	1309	1309	1313	$\delta(C_8H) + \nu(N_7C_8) + \nu(C_8N_9)$
1275 w, br	1270	1279	1298	1306<br>1295	1305<br>1298	$\nu(C_{1'}C_{2'}) - \nu(C_{2'}C_{3'}) + \delta(CH_{sugar})$
1238 w, br	1233	1239	1238	1215<br>1213	1213	$\nu(C_{1'}N_9) + \delta(C_8H) + \nu(C_4C_5)$
1178 w	1205	1198	very weak<br>1209	very weak<br>1182<br>1174	very weak<br>1182<br>1172	$\nu(C_1N_2) + \nu(N_3C_4) + \nu(C_4C_5) + \nu(C_4N_9) - \nu(C_5N_9)$ (ring breathing mode, nr 1-6)
1111 sh	1135	1136	1147	1157 (+)<br>1155 (-)	1153<br>1140	$\nu(C_1N_2) + \nu(C_{1'}O) + \nu(N_3C_4) - \nu(C_5N_7) + \delta(C_8H)$
	1117	1118	1111	1112<br>1110	1114<br>1106	$\nu(C_{1'}O) + \gamma(CH_{sugar})$
1078 s, br	1091	1097	1086	1078<br>1075	1089<br>1088	$\nu_{asym}(PO_3^{2-})$
976 s	1052	1057	1010	1005<br>1004	1011<br>1008	$\nu_{sym}(PO_3^{2-})$
802 w, br<br>777 w, br	888<br>883	893	873	881<br>880	889<br>869	$\nu(PO) + \gamma(CH_{sugar})$

[a] Peak intensities: s = strong, m = medium, w = weak, br = broad and sh = shoulder. [b] An uniform scaling factor of 0.9496 was applied to best match the 1800-640 cm^{-1} region of free 5'-GMP. [c] Notations used: ν = stretch, δ = in-plane angle bending, γ = out-of-plane angle bending, scr = scissoring, wag = wagging, asym = anti-symmetric, sym = symmetric, (+) = 5'-GMP vibrations in-phase, (-) = 5'-GMP vibrations out-of-phase, + = in-phase with previous vibration and - = out-of-phase with previous vibration. See Figure S1D for atom labels.

Table S2. Mean HPLC peak areas and standard deviations of the photoproducts from reactions of **1** (50 µM) and 5'-GMP (100 µM) monitored after 30 min of 420 nm irradiation.

Time	**1a**		**1b**		**1c**		**1d**		**1e**	
Days	Mean area (mAU*s)	Standard deviation (mAU*s)	Mean area (mAU*s)	Standard deviation (mAU*s)	Mean area (mAU*s)	Standard deviation (mAU*s)	Mean area (mAU*s)	Standard deviation (mAU*s)	Mean area (mAU*s)	Standard deviation (mAU*s)
0	95.15	0.74	207.89	4.93	70.51	2.08	-[a]	-	-	-
1	100.16	0.08	196.91	0.78	84.66	0.14	21.77	1.64	-	-
2	100.31	0.71	185.02	1.70	96.54	6.86	31.34	0.14	-	-
3	100.47	0.39	176.16	4.03	102.54	0.30	37.84	0.19	-	-
4	100.82	0.80	163.13	0.64	112.17	0.06	42.84	0.72	20.05	0.36
5	101.30	0.27	151.22	0.44	121.77	0.21	42.84	0.72	21.36	1.01
6	101.58	0.06	142.33	3.70	130.18	0.96	-	-	25.56	1.04
7	NaN[b]	NaN	NaN	NaN	NaN	NaN	NaN	NaN	NaN	NaN
8	NaN	NaN	NaN	NaN	NaN	NaN	NaN	NaN	NaN	NaN
9	101.43	0.21	109.40	0.77	154.52	0.15	-	-	24.77	0.06
10	102.02	0.13	104.46	0.81	159.19	0.16	-	-	25.35	0.06
11	NaN	NaN	NaN	NaN	NaN	NaN	NaN	NaN	NaN	NaN
12	102.41	0.20	94.22	0.34	165.17	0.16	-	-	26.39	0.08
13	NaN	NaN	NaN	NaN	NaN	NaN	NaN	NaN	NaN	NaN
14	102.36	0.77	82.89	0.19	167.55	0.87	-	-	25.79	0.28

[a] – implies no HPLC peak with an area above 5 mAU*s and height above 2 mAU was detected. [b] NaN implies no measurements were taken on this day.

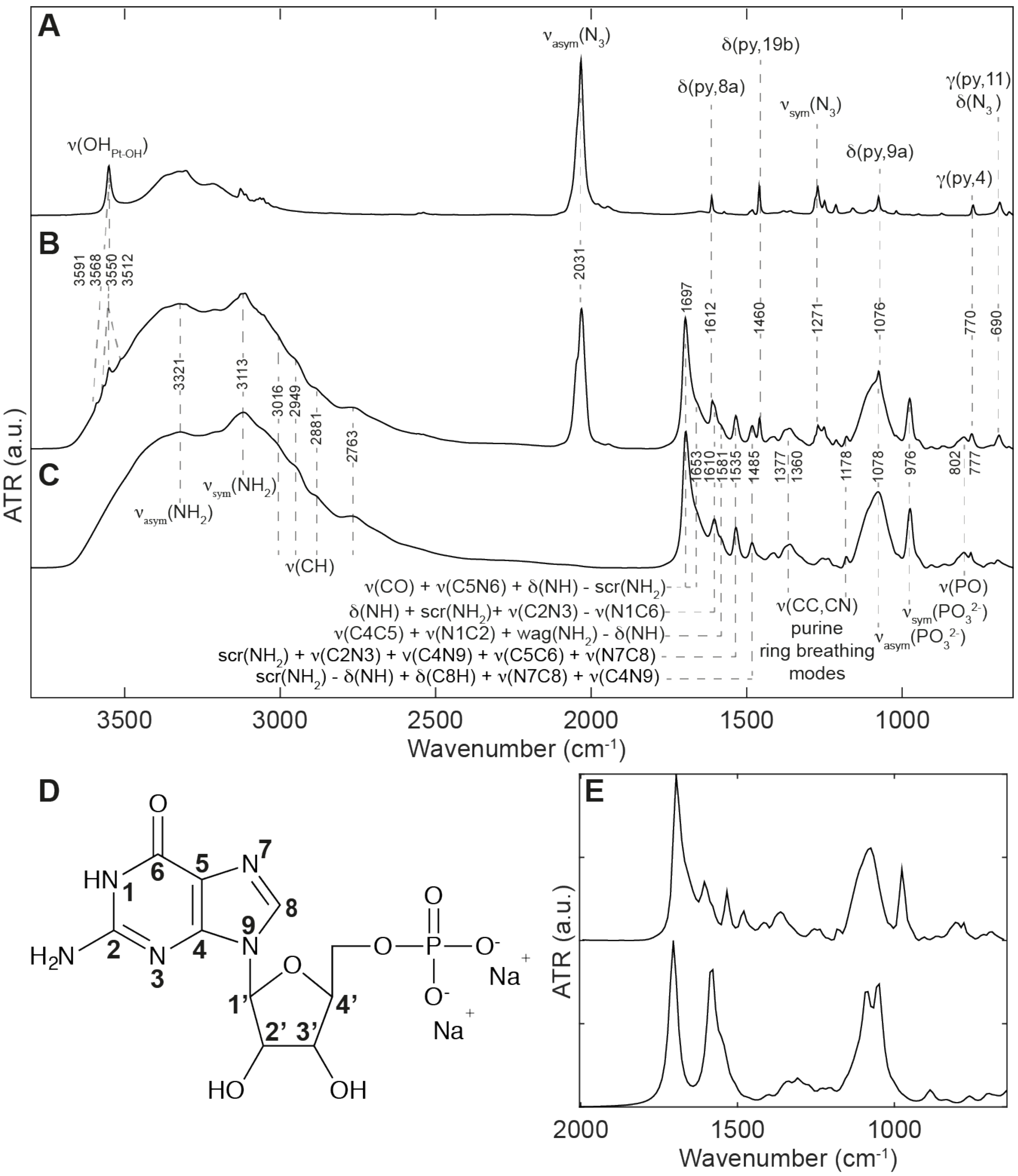

Figure S1. ATR-FTIR spectra including peak labels and assignments of **1** (A), **1** + 2 mol equiv. 5'-GMP (B), and 5'-GMP (C). D) Atom labels of 5'-GMP. E) ATR-FTIR of 5'-GMP (TOP) and computed infrared spectrum (BOTTOM) between 2000 – 640 cm^{-1}. Notations used: ν = stretch, δ = in-plane angle bending, γ = out-of-plane angle bending, sym = symmetric, asym = anti-symmetric, + = in-phase with previous vibration and - = out-of-phase with previous vibration. The labels for pyridine modes are given in Wilson's notations, as described previously.[1]

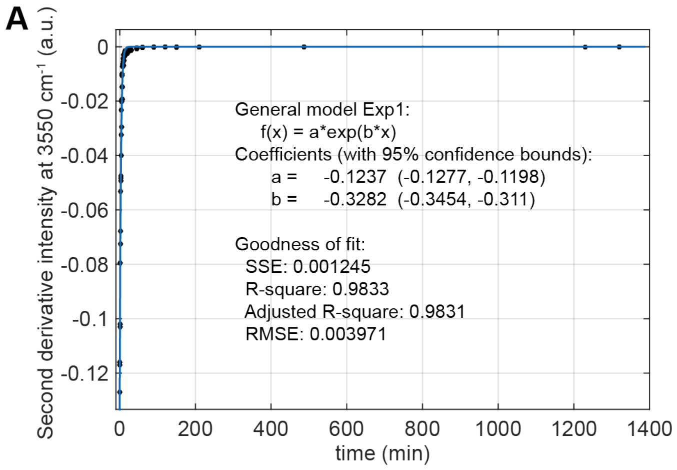

Figure S2. Second derivative ATR-FTIR intensity of ν($OH_{Pt\text{-}OH}$) at 3550 cm^{-1} of **1** over time (three experimental replicates, two measurements each replicate), upon irradiation at 420 nm. A) full time scale and 1st order exponential fit parameters. B) 0 – 20 minutes. Notations used: ν = stretch.

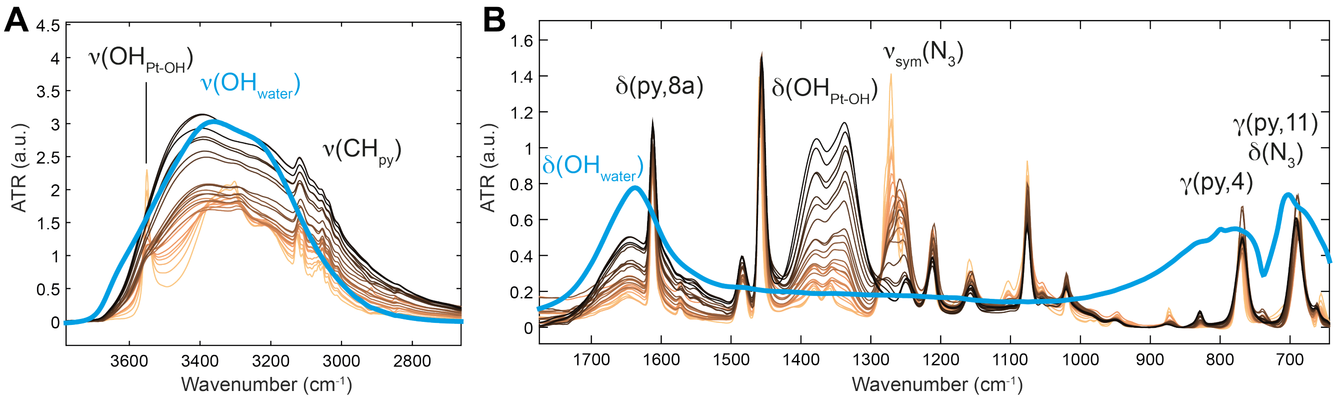

Figure S3. ATR-FTIR spectra of water (blue) and the photodecomposition of **1** (2 mM in water) under 420 nm irradiation between 3800 – 2600 cm^{-1} (A) and 1750 – 640 cm^{-1} (B). Colormap; light to dark = time 0 – time final, blue = water. Notations used: ν = stretch, δ = in-plane angle bending, γ = out-of-plane angle bending and sym = symmetric. The descriptions of pyridine modes are given in Wilson's notations, as described previously.[1]

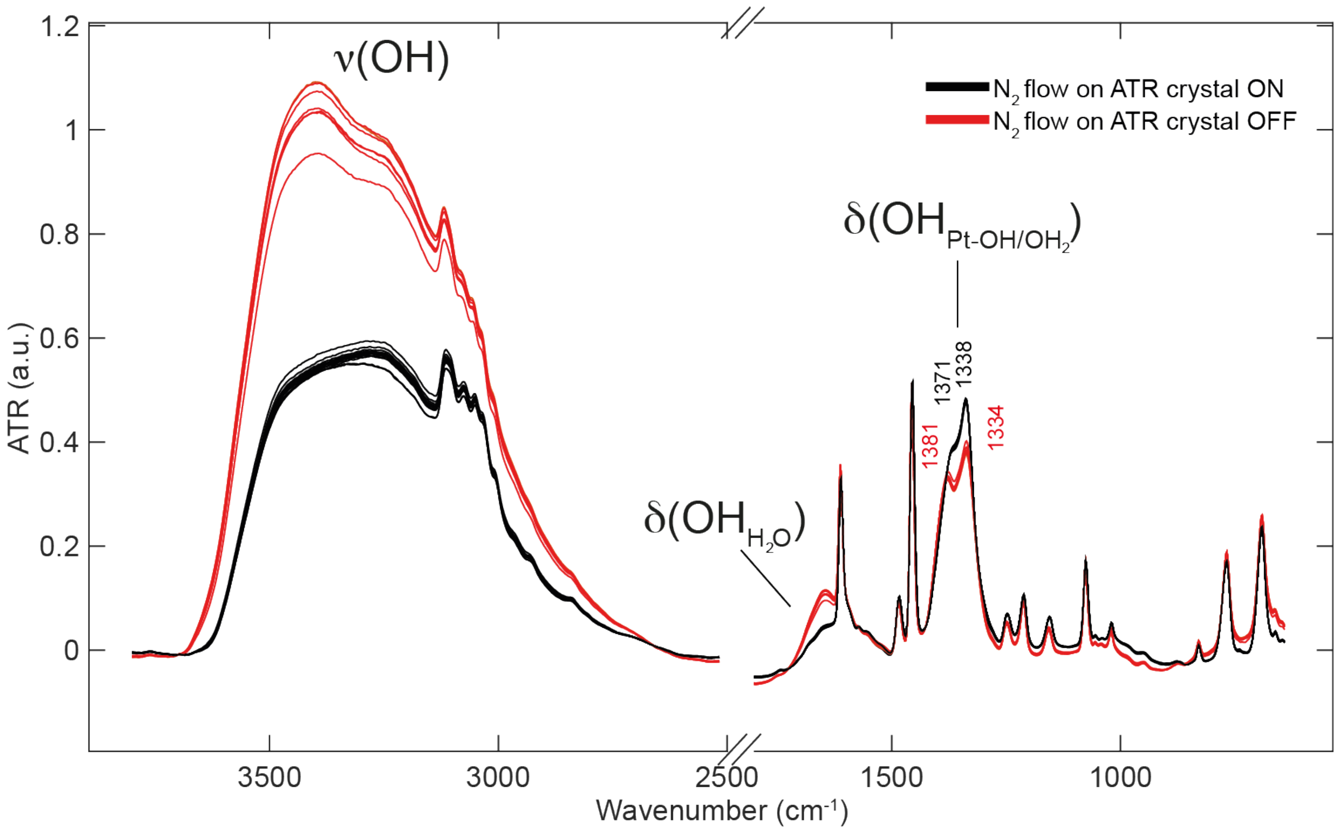

Figure S4. ATR-FTIR spectra of the final photoproduct of the photodecomposition of **1** (2 mM in water) under 420 nm irradiation. One scan per spectrum (±1 s) where the N_2 flow onto the ATR crystal/sample was switched ON and OFF with ±2 s intervals. Red = spectra taken without N_2 flow on ATR crystal, black = spectra taken with N_2 flow on ATR crystal. Notations used: ν = stretch and δ = in-plane angle bending.

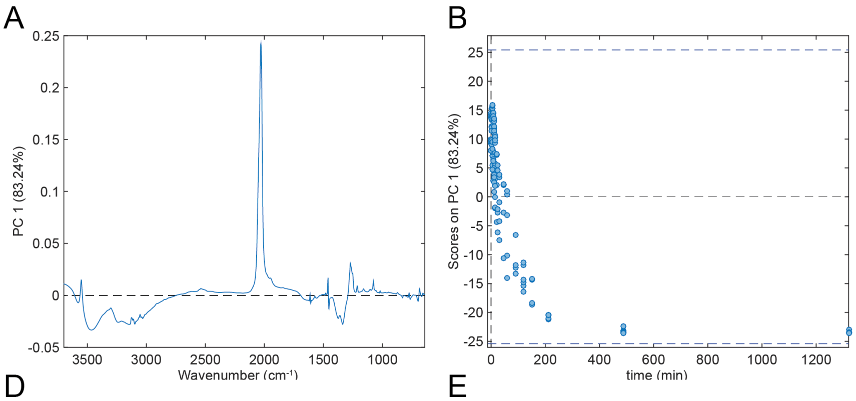

C

Principal Component Number	Eigenvalue of Covariance (X)	Variance Captured (%)	Total Variance Captured (%)	RMSEC	RMSECV
1	1.65E+02	83.24	83.24	0.1442	0.1689
2	1.12E+01	5.67	88.91	0.1173	0.1444
3	8.08E+00	4.07	92.98	0.09329	0.1375

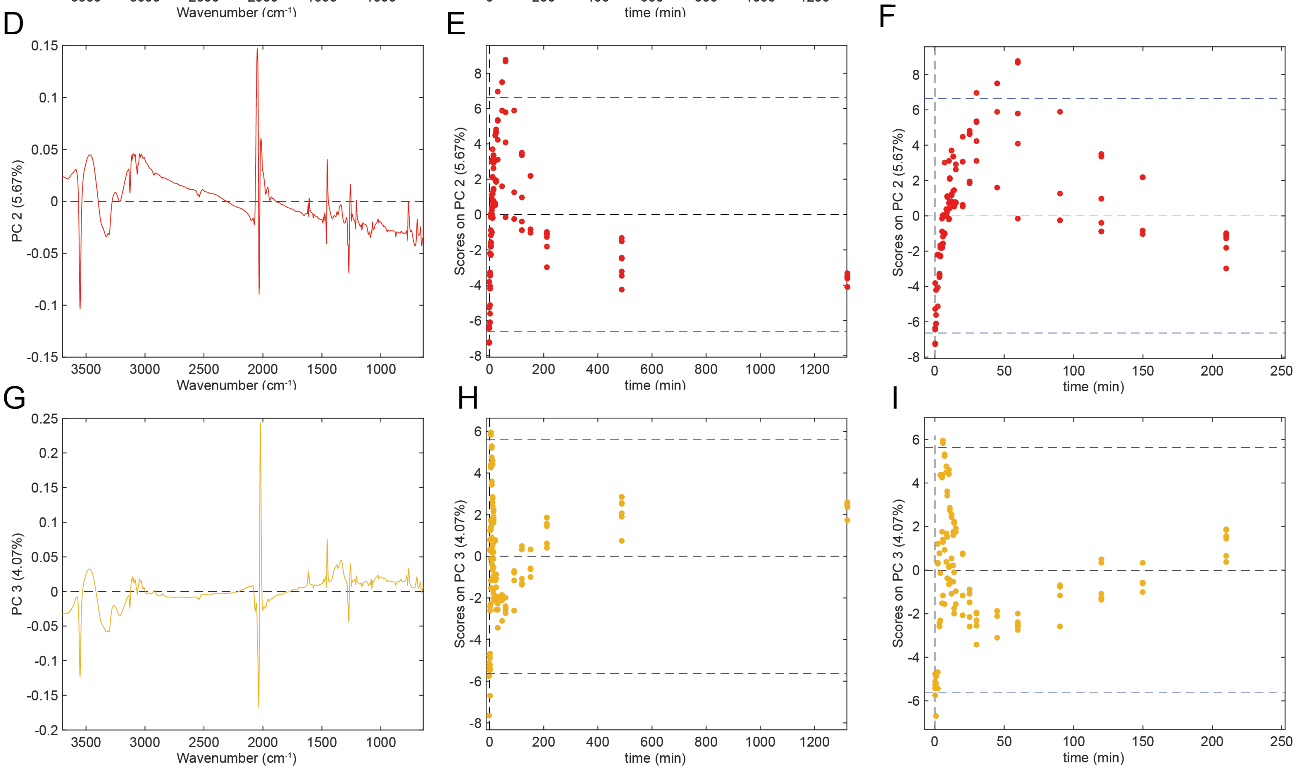

Figure S5. PCA analysis results for the photodecomposition of **1** in water under 420 nm irradiation by ATR-FTIR. A/D/G) Loading plots for PC 1/2/3. B/E/H) Scores vs. time plots of PC 1/2/3. C) Eigenvalues, variance captured, Root Mean Square Error of Calibration (RMSEC) and Root Mean Square Error of Cross Validation (RMSECV) of the Principal components (PC). F/I) Scores vs. time plots (0 – 250 min) of PC 2/3.

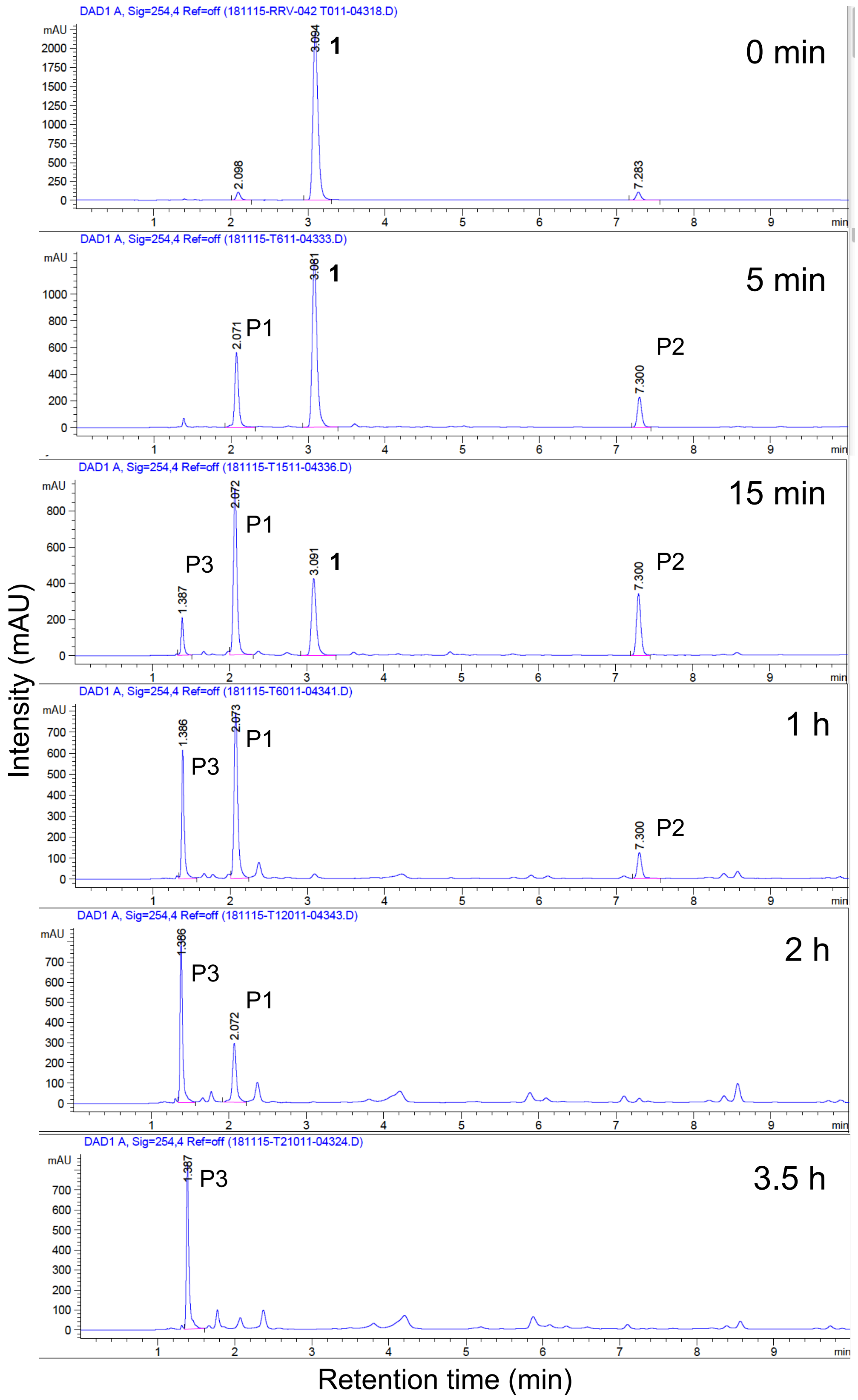

Figure S6. Analytical HPLC traces (254 nm) for the photodecomposition of **1** in water (2 mM) under 420 nm irradiation.

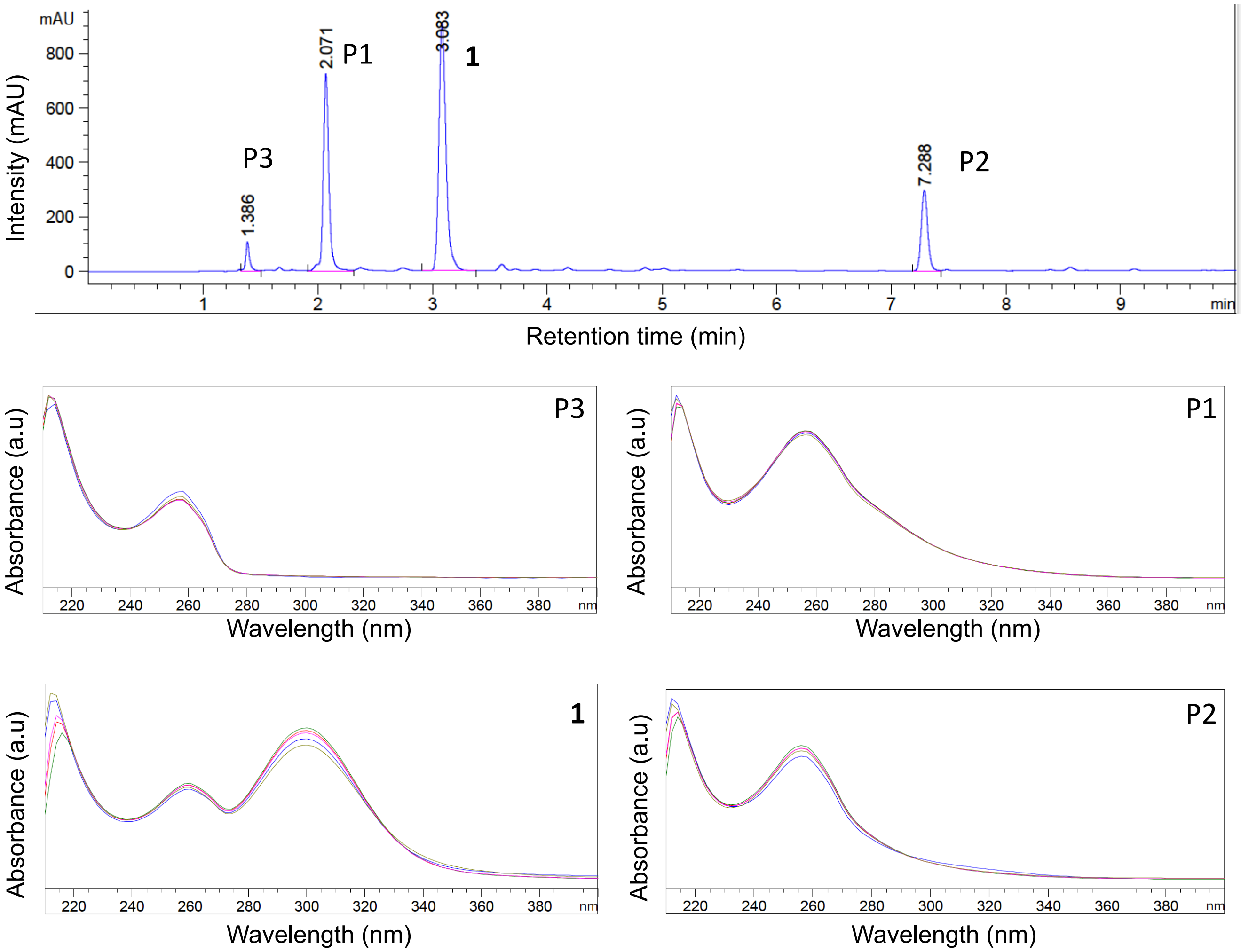

Figure S7. Analytical HPLC trace (254 nm) for **1** in water after 8 min of 420 nm irradiation, including UV-vis peak purity analysis spectra (5 spectra per integration) for **1** and its photoproducts P1, P2 and P3.

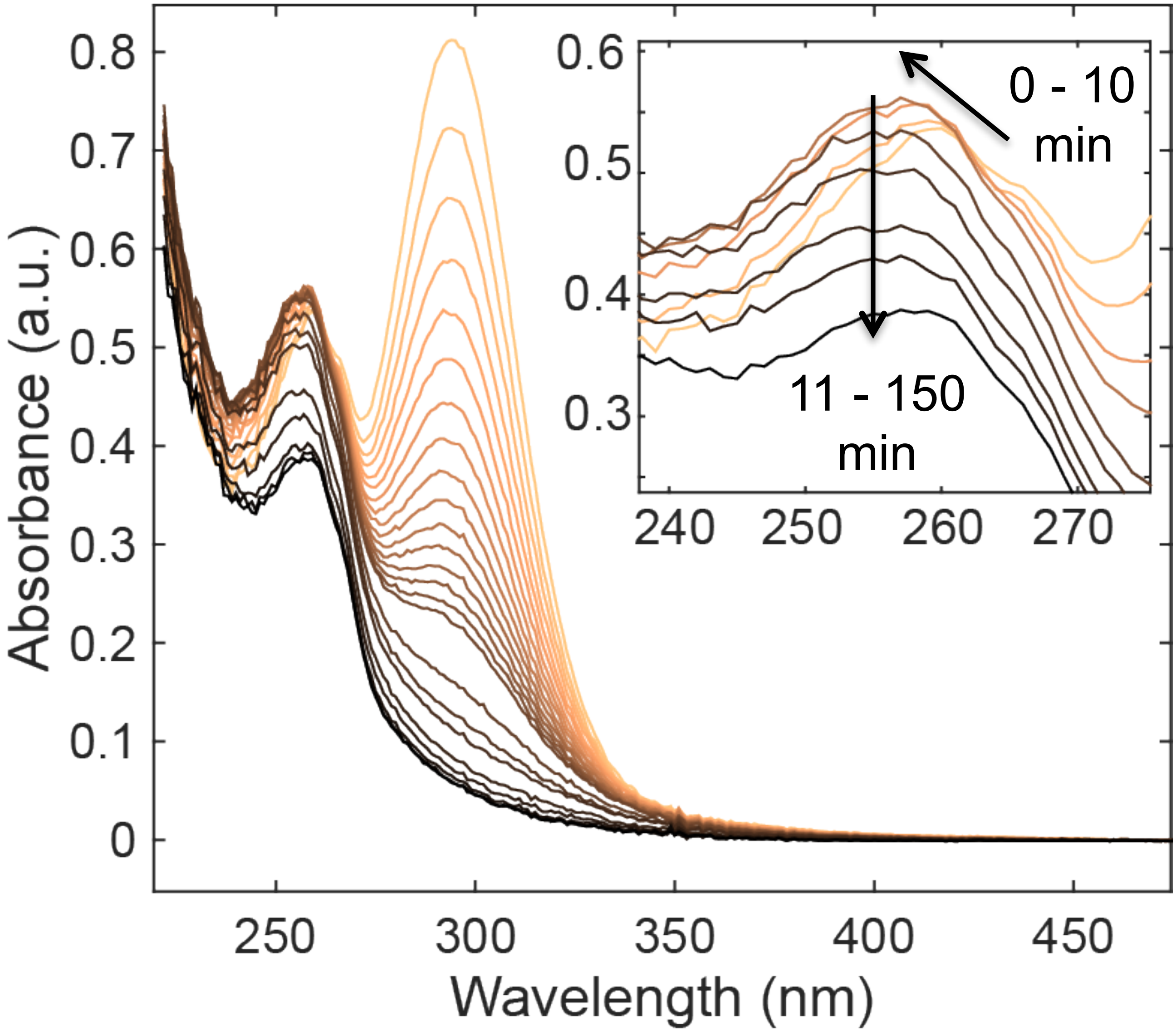

Figure S8. UV-vis spectra for the photodecomposition of **1** in water (50 μM) under 420 nm irradiation. Colormap; light to dark = time 0 – time final.

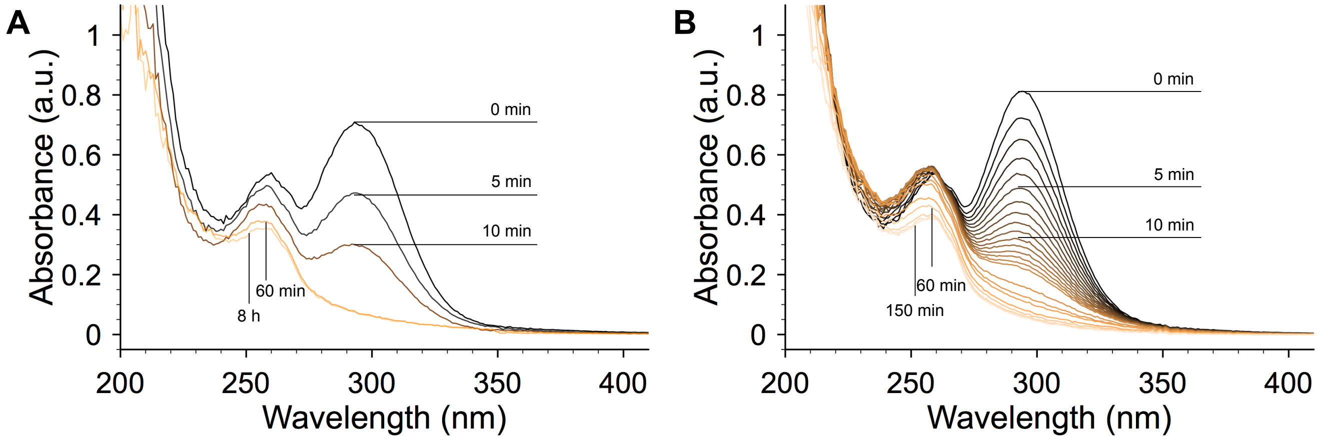

Figure S9. UV-vis spectra of the photodecomposition of **1** in water under 420 nm irradiation. A) irradiation of 2 mM solution of **1** in water, diluted to 50 μM. B) irradiation of 50 μM solution of **1**.

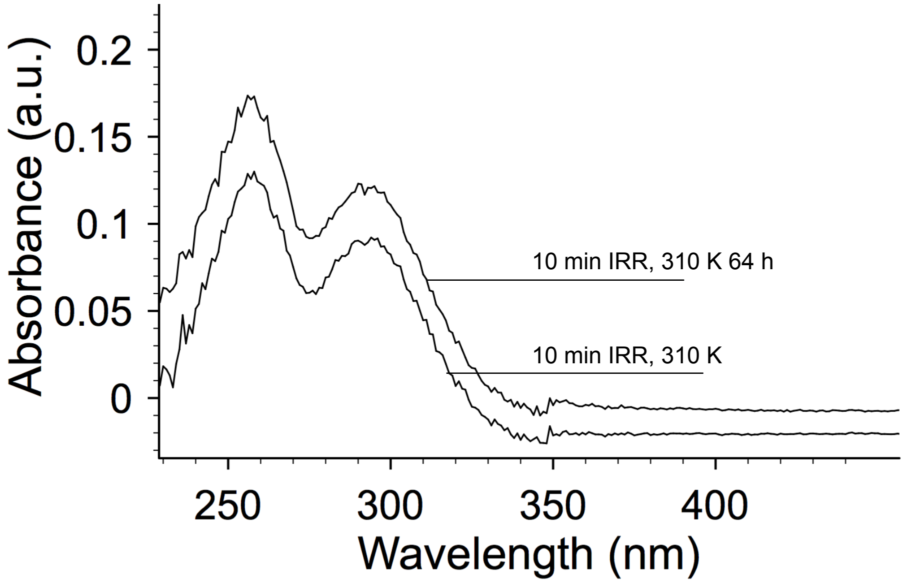

Figure S10. UV-vis spectra of **1** after 10 min of 420 nm irradiation at 310 K and a further 64 h at 310 K.

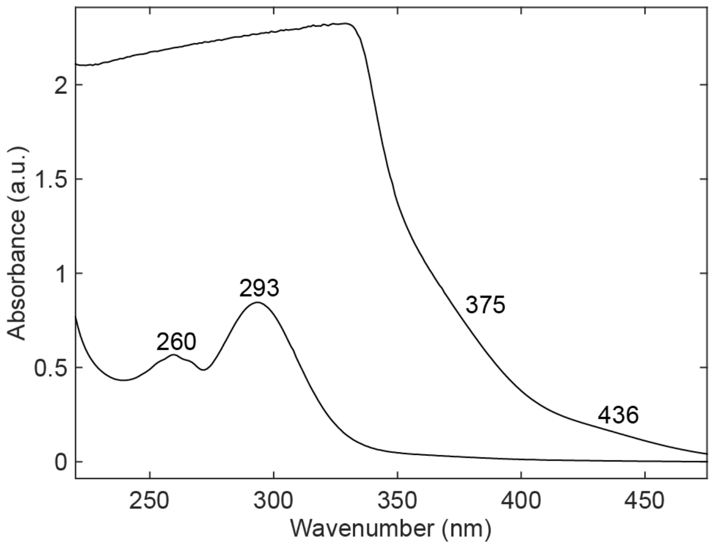

Figure S11. UV-vis spectra of **1** in acetonitrile (50 μM, bottom and 2 mM, top).

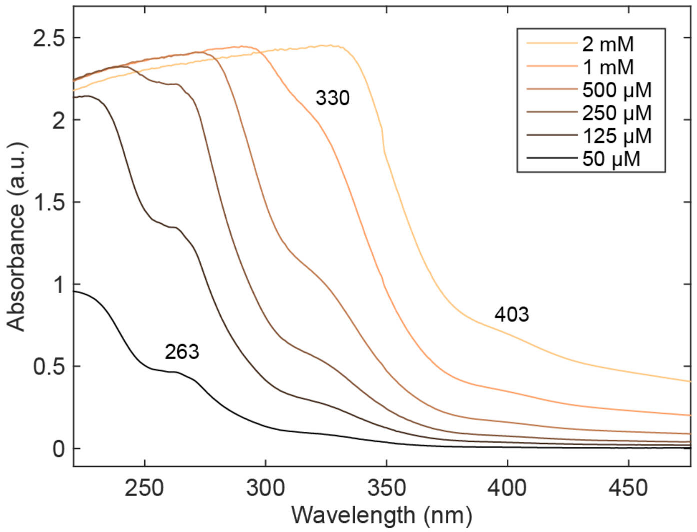

Figure S12. UV-vis spectra of **2** in acetonitrile.

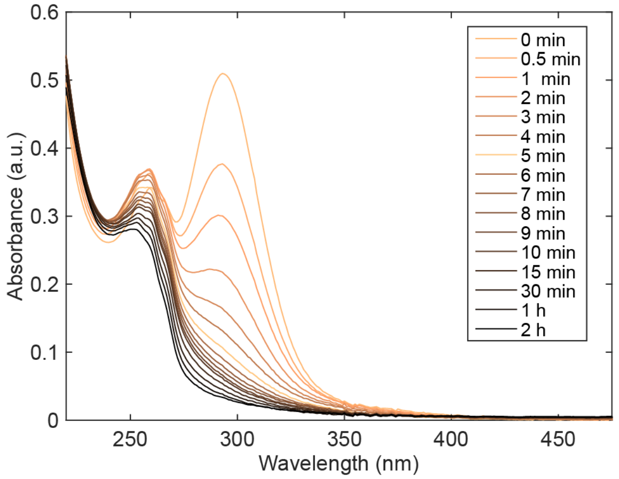

Figure S13. UV-Vis spectra of **1** in acetonitrile (50 μM) upon 310 nm irradiation.

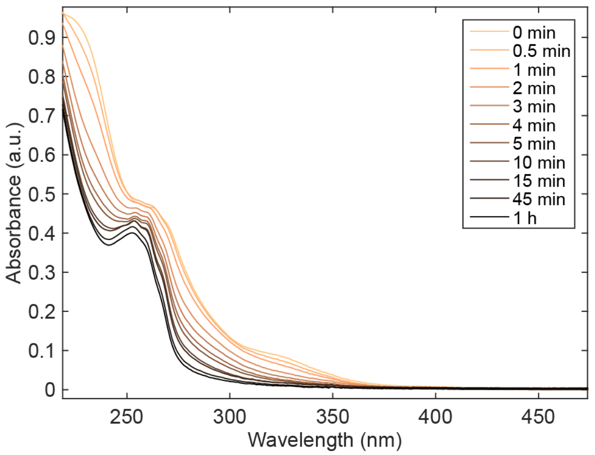

Figure S14. UV-vis spectra of **2** in acetonitrile (50 μM) upon 310 nm irradiation.

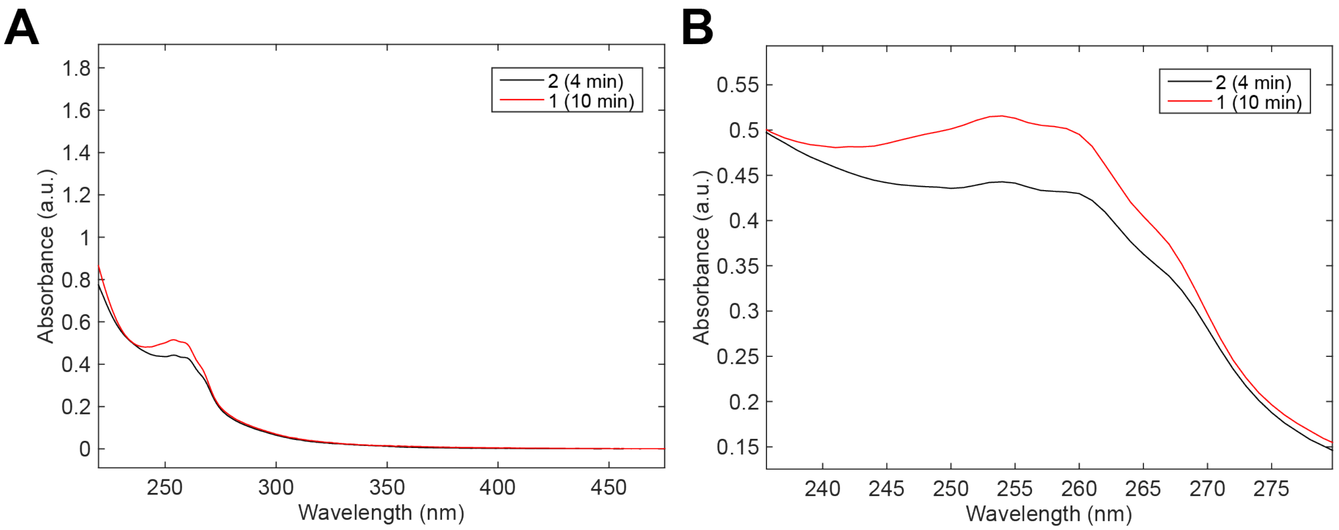

Figure S15. UV-vis spectra of **1** and **2** in acetonitrile upon 310 nm irradiation (10 min for **1** and 4 min for **2**).

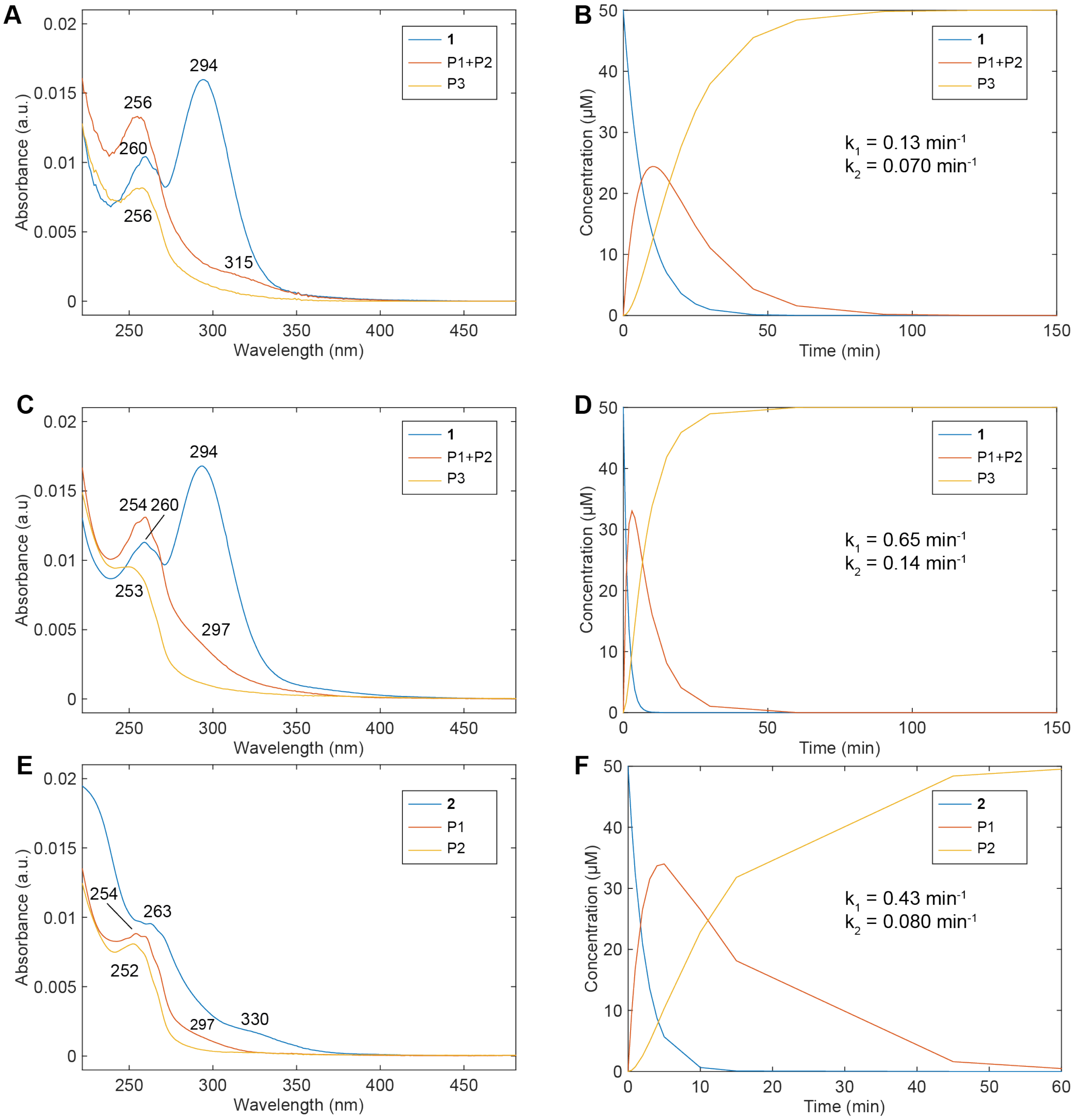

Figure S16. Deconstructed UV-vis spectra and concentration profiles of the photodecomposition of **1** and **2** and their associated products obtained through kinetic fitting (A>B>C) of the experimental data by MCR-ALS. A) Deconstructed spectra of **1** in water under 420 nm irradiation. C) Deconstructed spectra of **1** in acetonitrile under 310 nm irradiation. E) Deconstructed spectra of **2** in acetonitrile under 310 nm irradiation. B/D/F) Concentration profiles including rate constants k_1 and k_2.

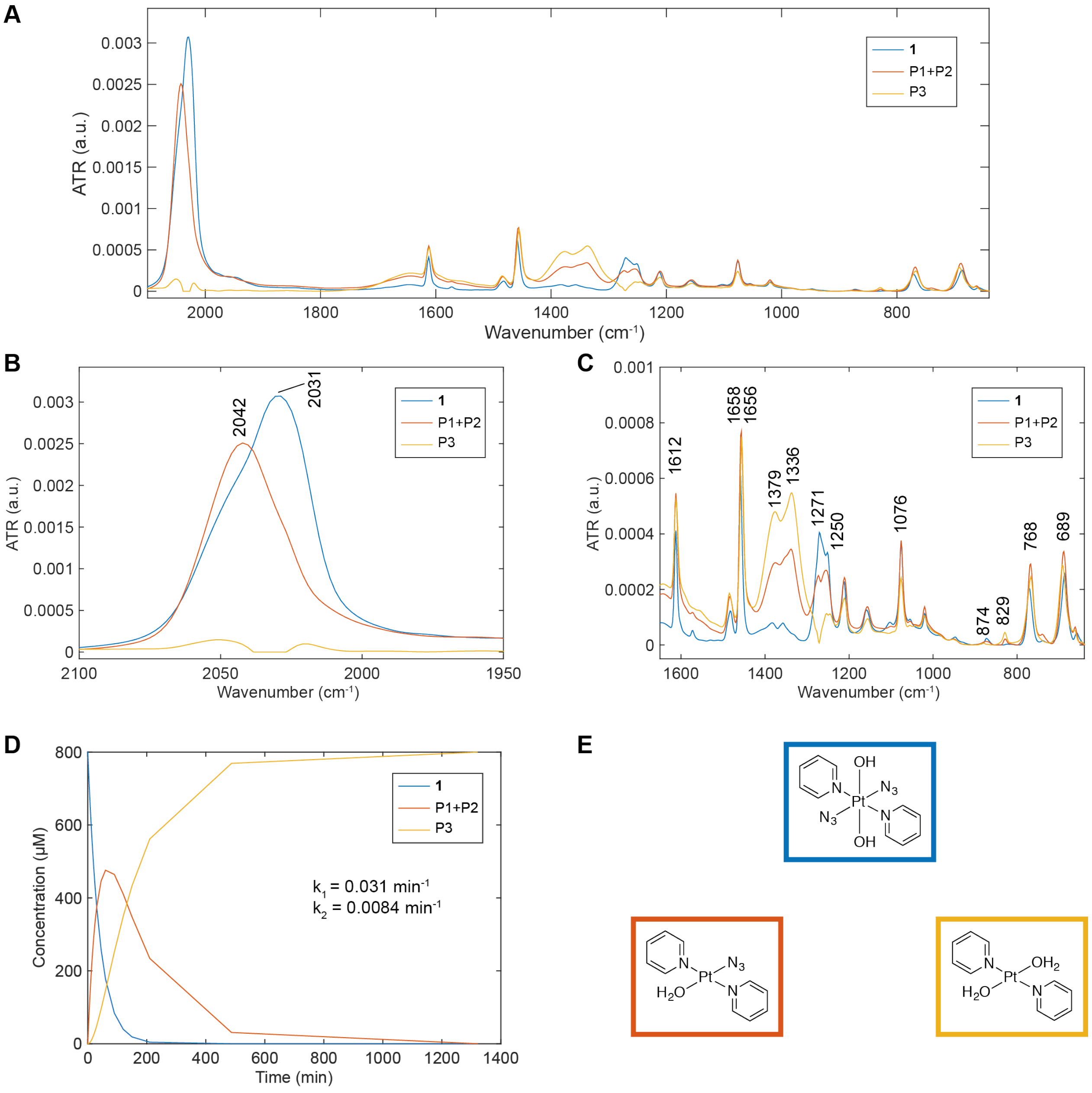

Figure S17. Deconstructed ATR-FTIR spectra and concentration profiles of the photodecomposition of **1** (420 nm irradiation in water) and its associated products obtained through kinetic fitting (A (**1**) > B (P1+P2) > C (P3)) of the experimental data by MCR-ALS. A) Deconstructed spectra, 2100 – 640 cm^{-1}. B) Deconstructed spectra, 2100 – 1950 cm^{-1}, highlighting $\nu_{asym}(N_3)$ peak labels. C) Deconstructed spectra, 1650– 640 cm^{-1}, including selected peak labels. D) Concentration profile including rate constants k_1 and k_2. E) Proposed structures for the three deconstructed spectra; blue = **1**, red = P1+P2 and yellow = P3.

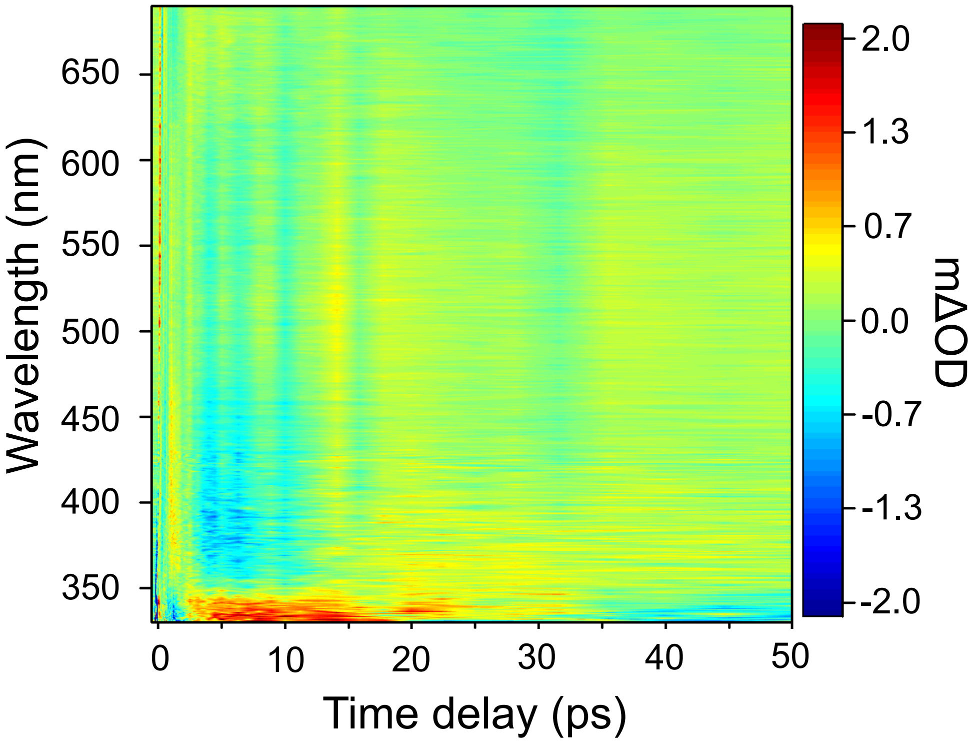

Figure S18. TAS residual plot. The average deviation is ~5%, however we note that at wavelengths shorter than 350 nm, the deviation is higher. This is due to the increased noise in signal as the probe intensity is at its weakest.

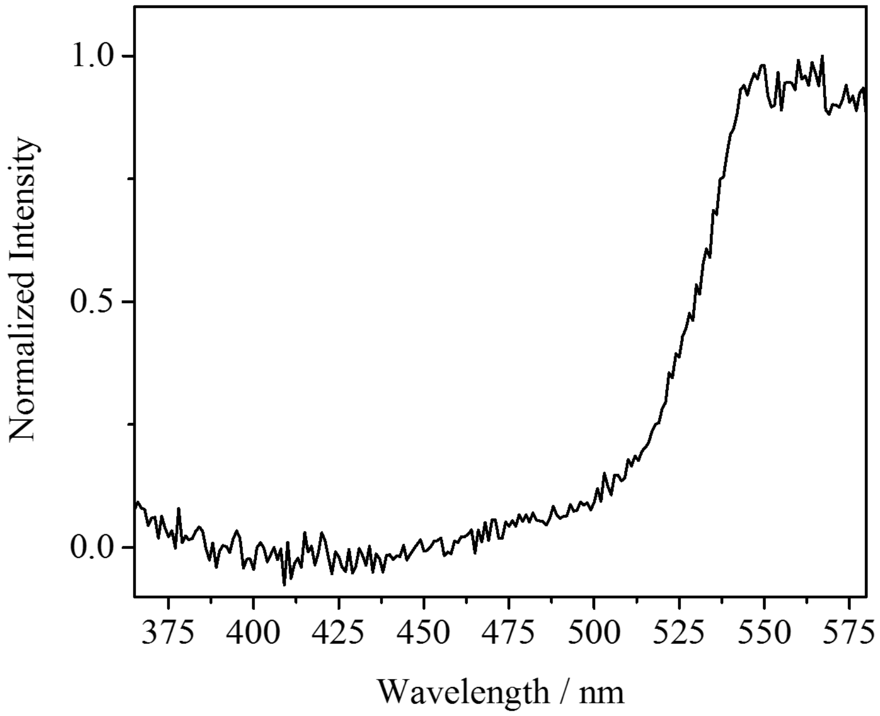

Figure S19. Emission spectrum of **1** in acetonitrile under 310 nm excitation.

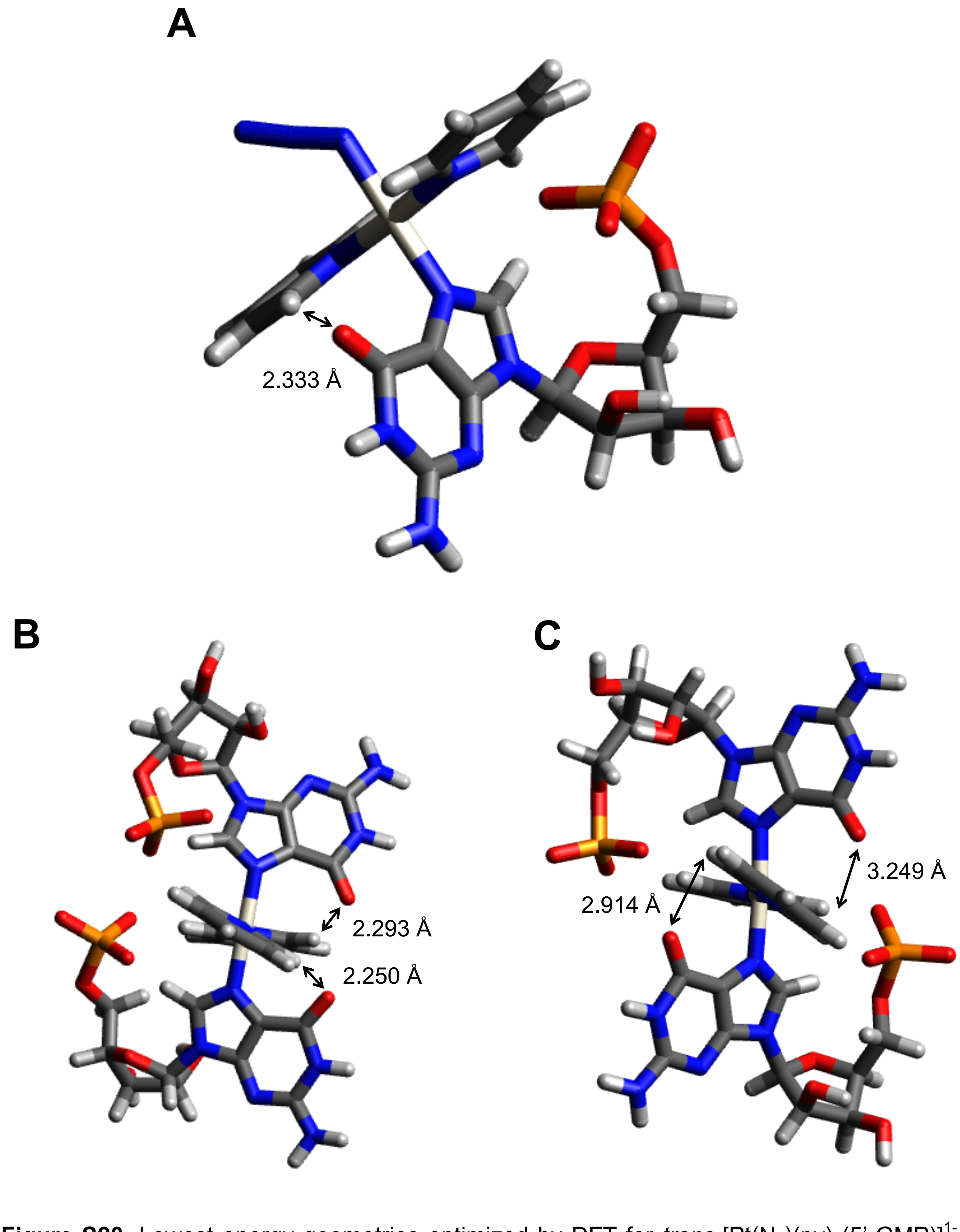

Figure S20. Lowest energy geometries optimized by DFT for *trans*-$[Pt(N_3)(py)_2(5'\text{-}GMP)]^{1-}$ (A), *trans*-$[Pt(py)_2(5'\text{-}GMP)_2]^{2-}$ (B) and *trans*-$[Pt(py)_2(5'\text{-}GMP)_2]^{2-}$ (C).

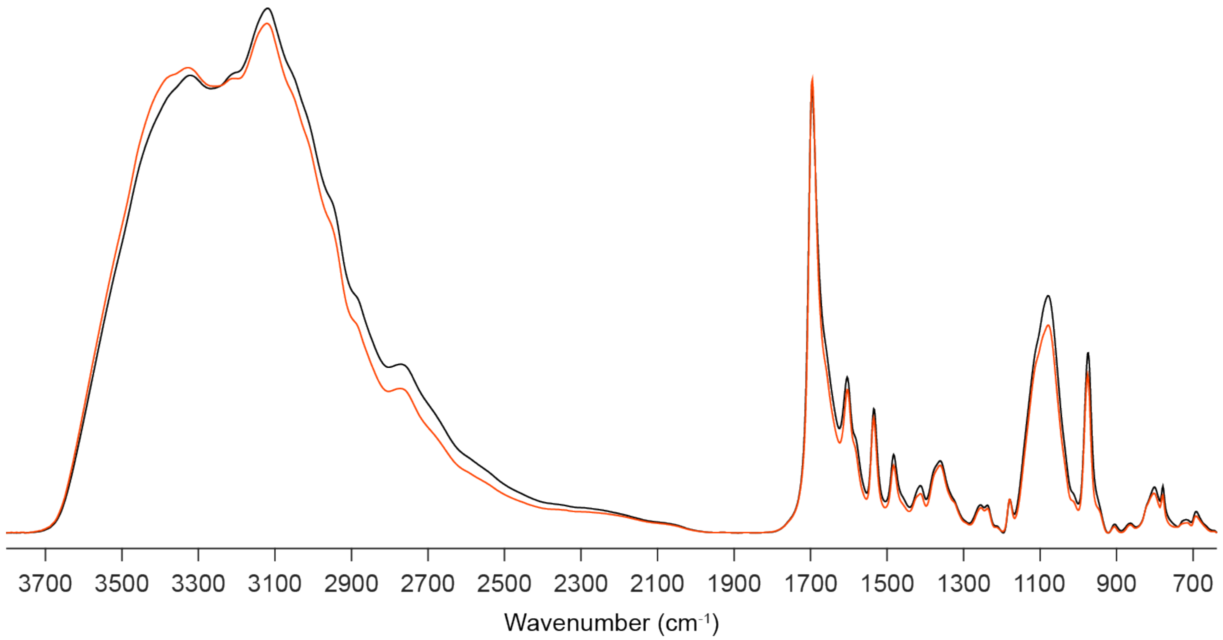

Figure S21. ATR-FTIR of 5'-GMP before (black) and after 22 h (red) of 420 nm irradiation.

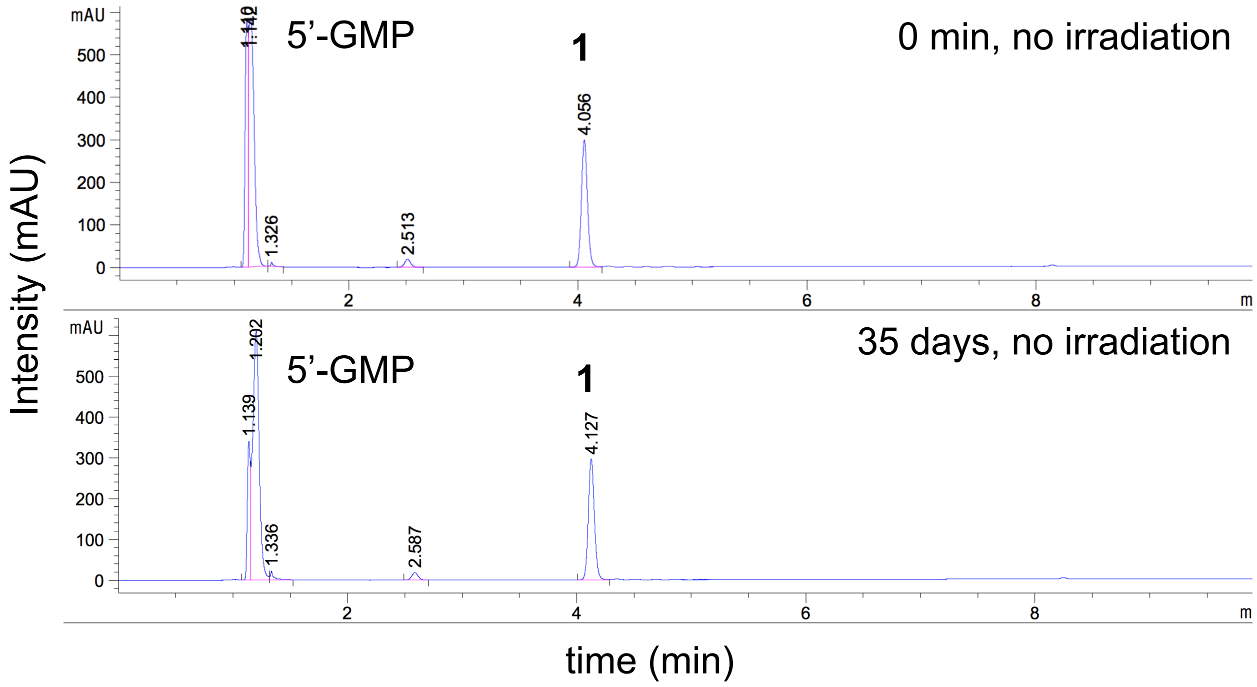

Figure S22. Analytical HPLC traces for reaction of **1** with 5'-GMP (1:2) in the dark at 0 min and after 35 d.

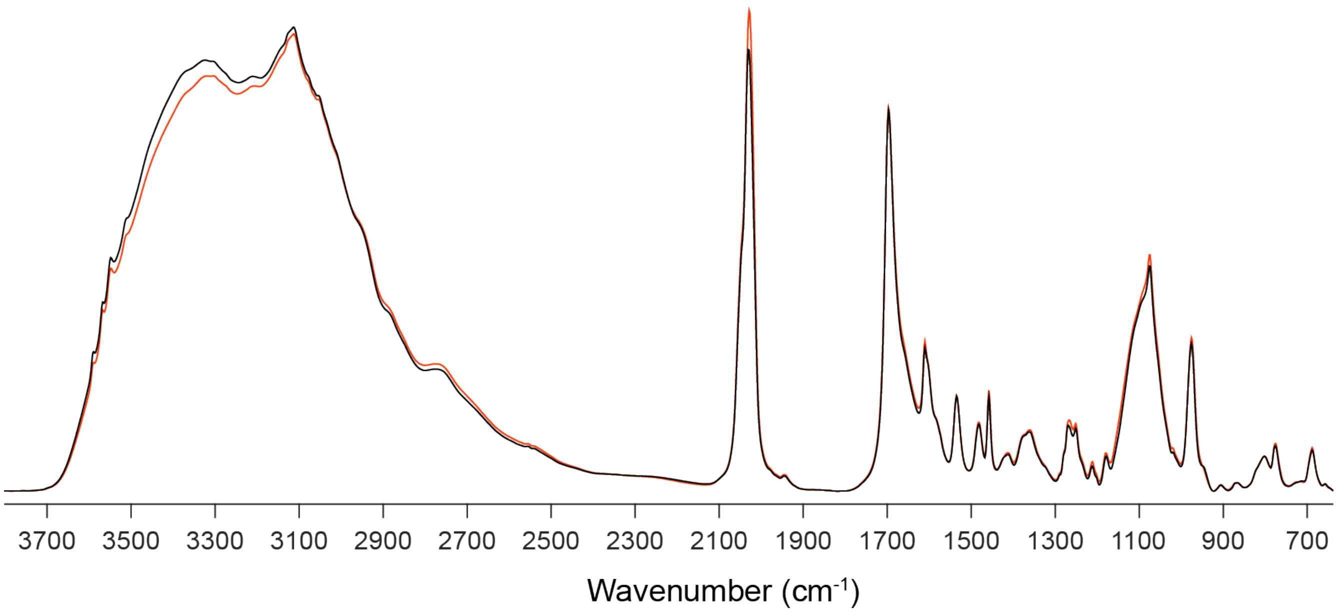

Figure S23. ATR-FTIR of **1** with 5'-GMP (1:2) in the dark immediately after mixing (black) and after 35 d (red).

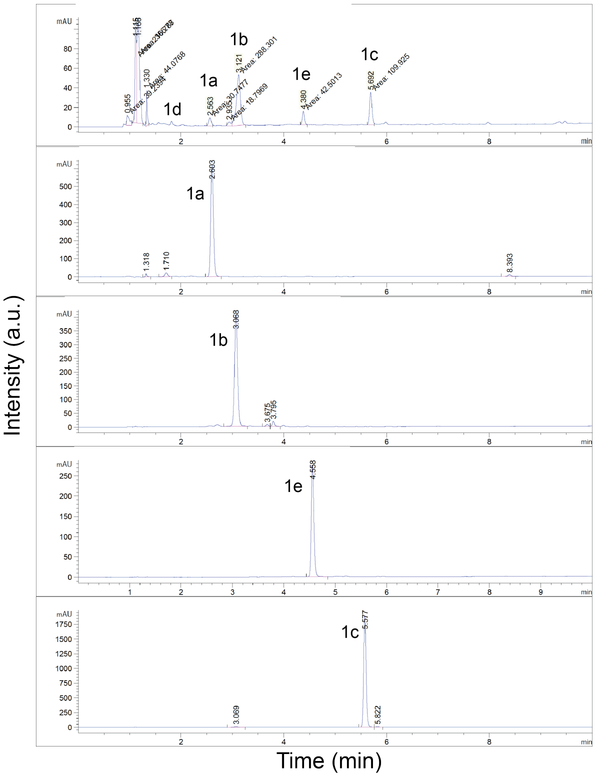

Possible species	LCMS	HRMS	Calcd.	ATR-FTIR
**1a**				
$Pt(III)(py)_2(OH)_2]^+$	387.1	-	387.05	Figure S26
$[Pt(N_3)(py)_2(OH)_2$(5'-GMP-2Na+2H)] + Na^+	815.2		815.11	
$[Pt(N_3)(py)_2(OH)_2$(5'-GMP)] + K^+	875.2		875.05	
$[Pt(N_3)(py)_2(OH)_2$(5'-GMP)] + K^+ + H_2O	893.2		893.06	
**1b**				
$[Pt(IV)(py)_2(N_3)$(5'-GMP-2Na)]$^+$	756.0	-		
	757.0			
	755.3			
$[Pt(IV)(py)_2(N_3)$(5'-GMP-2Na)(OH)] + H^+ or $[Pt(II)(py)_2(N_3)$(5'-GMP-2Na-H+OH)]$^{1-}$ + $2H^+$	774.0	774.1355	774.11 (100.0%)	Not stable
	773.0	773.1329	773.11 (97.4%)	
	775.0	775.1360	775.11 (74.6%)	
		776.1376	776.11 (21.6%)	
**1e**				
$[Pt(II)(py)_2$(5'-GMP-2Na+2H)$_2]^{2+}$	539.6	-	539.58 (100.0%)	Figure S26
	539.2		539.08 (97.4%)	
	540.2		540.08 (74.6%)	
	540.8		540.58 (24.2%)	
	541.2		541.08 (21.2%)	
**1c**				
$[Pt(II)(py)_2(N_3)$(5'-GMP-2Na+2H)]$^+$	758.1	758.1166	758.12 (100.0%)	Figure S26
	757.1	757.1146	757.11 (97.4%)	
	759.2	759.1172	759.12 (74.6%)	
	760.1	760.11	760.12 (21.6%)	
		761.1183	761.12 (21.2%)	

Figure S24. Analytical HPLC traces (254 nm) for the photoinduced reaction of **1** with 5'-GMP (1:2) under 420 nm irradiation after one hour (top), separated fractions (1a, 1b, 1c and 1e) and tabulated MS data including possible species.

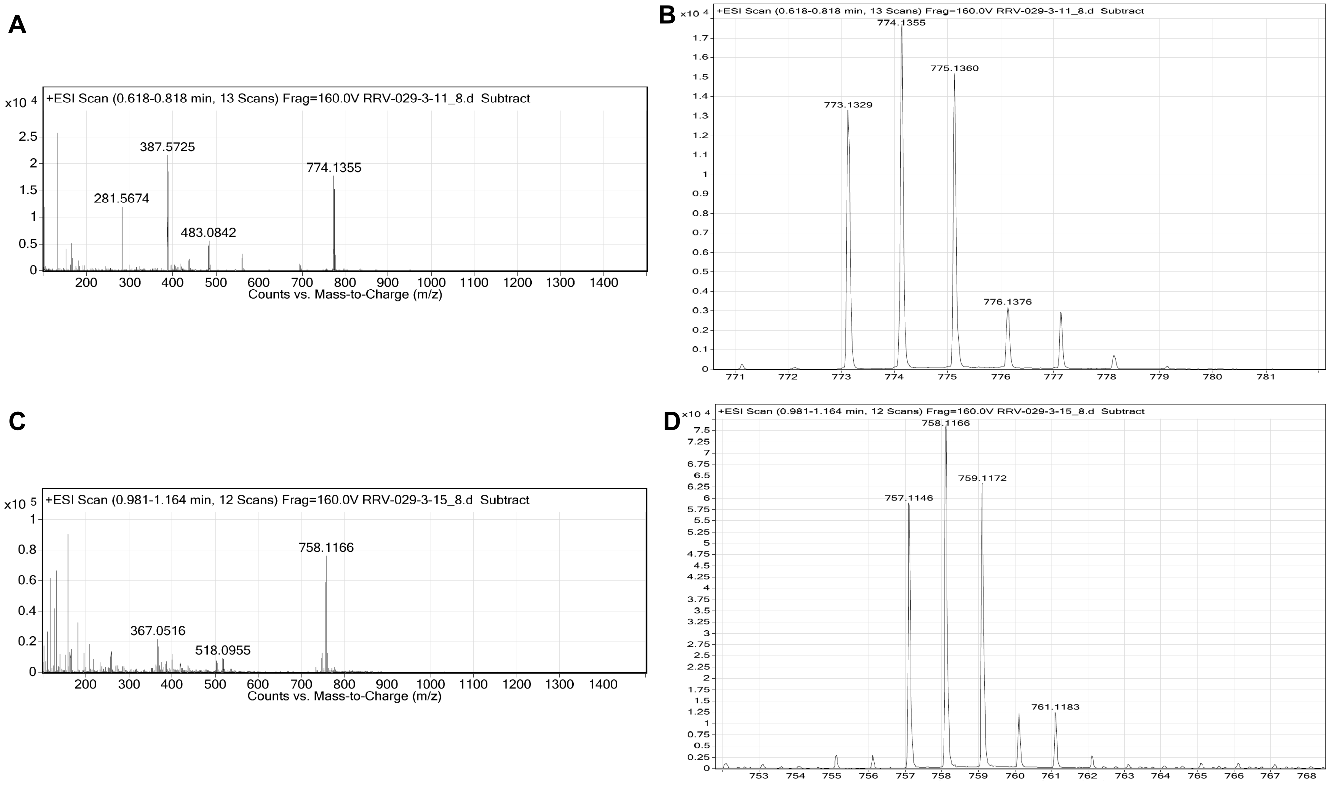

Figure S25. HRMS scans and *m/z* expansions of separated fractions 1b (A, B, *trans*-$[Pt(N_3)(py)_2(5'\text{-GMP-2Na})+OH+H]^+$) and 1c (C, D, *trans*-$[Pt(N_3)(py)_2(5'\text{-GMP-2Na+2H})]^+$) formed during the irradiation of **1** with 5’-GMP (1:2) under 420 nm irradiation.

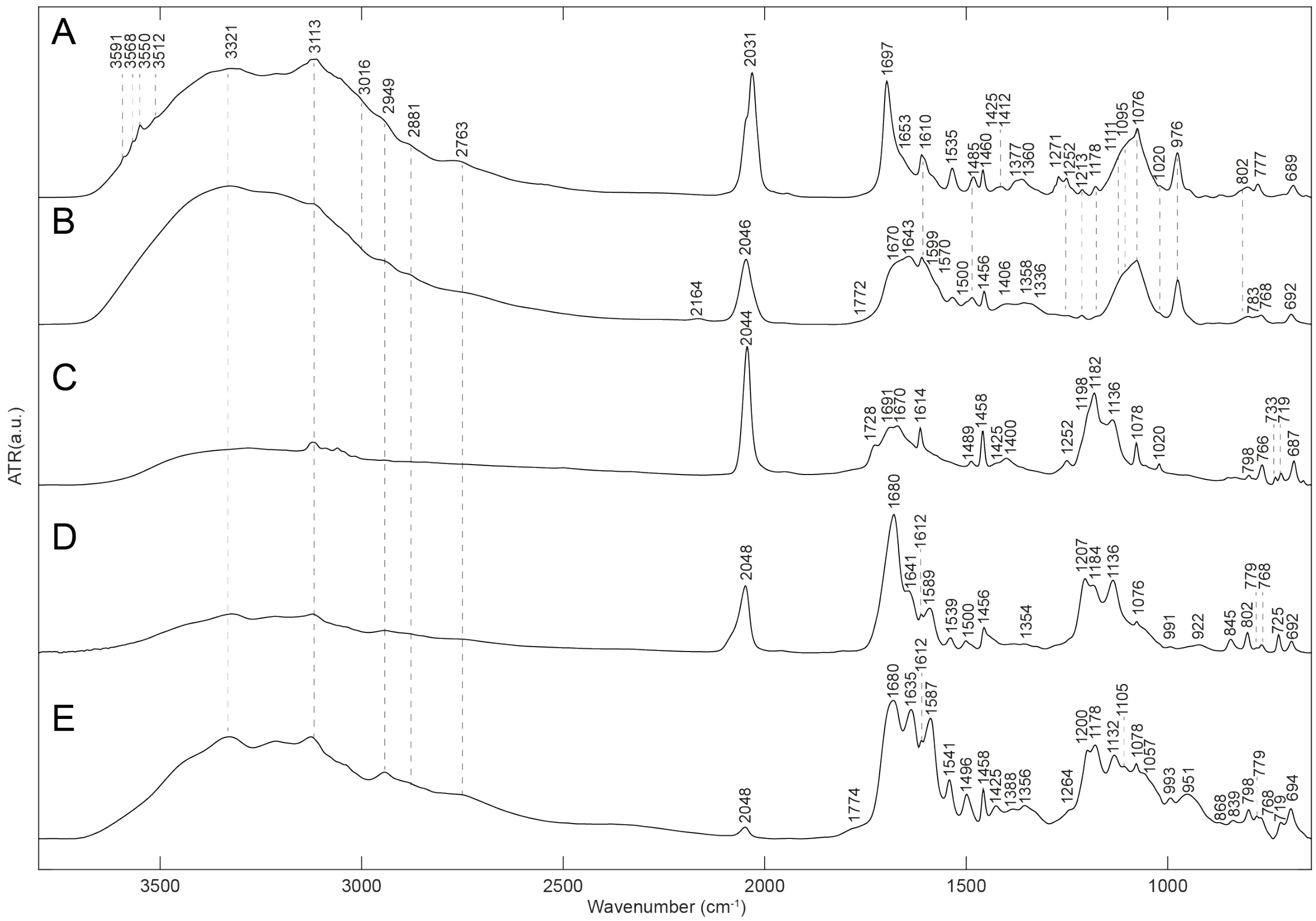

Figure S26. ATR-FTIR of **1** with 5'-GMP (1:2) under 420 nm irradiation, including peak labels. A) Dark, before irradiation. B) After 120 min of irradiation. C) Separated photoproduct 1a, *trans*-$[Pt(N_3)(OH)_2(py)_2(5'\text{-GMP})]$. D) Separated photoproduct 1c, *trans*-$[Pt(N_3)(py)_2(5'\text{-GMP})]$. E) Separated photoproduct 1e, *trans*-$[Pt(py)_2(5'\text{-GMP})_2]$.

References

[1] a) E. B. Wilson, *Phys. Rev.* **1934**, *45*, 706-714; b) R. R. Vernooij, T. Joshi, E. Shaili, M. Kubeil, D. R. T. Appadoo, E. I. Izgorodina, B. Graham, P. J. Sadler, B. R. Wood, L. Spiccia, *Inorg. Chem.* **2016**, *55*, 5983-5992.
